# An integrated calcium imaging processing toolbox for the analysis of neuronal population dynamics

**DOI:** 10.1371/journal.pcbi.1005526

**Published:** 2017-06-07

**Authors:** Sebastián A. Romano, Verónica Pérez-Schuster, Adrien Jouary, Jonathan Boulanger-Weill, Alessia Candeo, Thomas Pietri, Germán Sumbre

**Affiliations:** 1 Ecole Normale Supérieure, PSL Research University, CNRS, Inserm, Institut de Biologie de l'ENS, IBENS, Paris, France; 2 Instituto de Investigación en Biomedicina de Buenos Aires – CONICET – Partner Institute of the Max Planck Society, Buenos Aires, Argentina; Université Paris Descartes, Centre National de la Recherche Scientifique, FRANCE

## Abstract

The development of new imaging and optogenetics techniques to study the dynamics of large neuronal circuits is generating datasets of unprecedented volume and complexity, demanding the development of appropriate analysis tools. We present a comprehensive computational workflow for the analysis of neuronal population calcium dynamics. The toolbox includes newly developed algorithms and interactive tools for image pre-processing and segmentation, estimation of significant single-neuron single-trial signals, mapping event-related neuronal responses, detection of activity-correlated neuronal clusters, exploration of population dynamics, and analysis of clusters' features against surrogate control datasets. The modules are integrated in a modular and versatile processing pipeline, adaptable to different needs. The clustering module is capable of detecting flexible, dynamically activated neuronal assemblies, consistent with the distributed population coding of the brain. We demonstrate the suitability of the toolbox for a variety of calcium imaging datasets. The toolbox open-source code, a step-by-step tutorial and a case study dataset are available at https://github.com/zebrain-lab/Toolbox-Romano-et-al.

This is a *PLOS Computational Biology* Software paper.

## Introduction

Brain function relies on the interaction of large neuronal populations. Anomalies of these complex neuronal circuits are associated with diverse brain disorders[1]. Therefore, to understand brain function both in health and disease, it is necessary to explore the activity dynamics of neuronal networks. Recent advances in optical methods and optogenetics provide unprecedented possibilities for functional imaging of large neuronal populations[2–5], and even whole brains[6,7], with high spatial (e.g., single-neuron) resolution. These imaging datasets can be analyzed to reveal the responses of spatially distributed neuronal populations to sensory, motor, or task variables[5,8–11].

In contrast to the important experimental advances in imaging techniques, computational analysis tools are still incipient, consisting of processing pipelines that widely vary across labs, leading to poor standardization[12,13]. Few integrated software packages are currently available, and they all focus on different aspects of the initial data pre-processing stages of the analysis workflow[14–16]. Nevertheless, the study of large neuronal circuits imposes new challenges to the processing and analysis of the complex, high-dimensional datasets typically acquired. In order to make sense of neuronal population dynamics, a popular approach used is based on statistical methods of dimensionality reduction[17]. These methods project the high-dimensional data into a low-dimensional space that preserves or reveals underlying features of the data. Remarkably, they have been applied to expose organizing principles of neuronal networks dynamics[18–20] and to detect neuronal assemblies[9,21–23]. The identification of neuronal assemblies (i.e., neuronal subsets that show correlated activity) is a significant step towards a systemic understanding of neuronal circuits, since they can reflect functional processing modules[2]. Importantly, neuronal assemblies are not rigid structures with unique functions. On the contrary, they are dynamic, multifunctional, adaptive and overlapping units[3]. For instance, a neuronal assembly could perform a computation at a given time, but at a different time point, a subgroup of this assembly could be part of a different assembly with a different functional role. Hence, neurons could belong to multiple assemblies (i.e., non-exclusive assemblies) and play diverse roles in different brain processes. Indeed, it is in neuronal assemblies' flexible and distributed nature wherein lies one of the major difficulties in identifying them[4].

For a recent study, we developed an algorithm to characterize the spatio-temporal structure of neuronal activity patterns observed in large neuronal populations imaged using two-photon microscopy[9]. The framework applies dimensionality reduction and clustering techniques for the analysis of calcium imaging datasets, outperforming other traditional algorithms in the effective detection of neuronal assemblies embedded within neuronal networks[9]. This method is able to detect non-exclusive assemblies that are engaged and disengaged on a moment-to-moment basis, compatible with the distributed and dynamic nature of brain processing[24].

Here, we present a computational toolbox that integrates this method for detecting neuronal assemblies into a complete data processing pipeline designed for the comprehensive analysis of fluorescence imaging data, from the raw images to interpretable results on neuronal population dynamic. It consists of modules for video pre-processing, morphological image segmentation into regions of interest (ROIs) corresponding to single neurons, extraction of fluorescence signals, analysis of ROI responses to stimulus and/or behavioral variables, detection of assemblies of ROIs, exploratory analysis of network dynamics and the automatic generation of surrogate shuffled datasets to act as controls for statistical purposes. Typically, the full protocol can be completed on a workstation computer in ~1 hour, depending on the size of the dataset.

The different processing algorithms that we now implement in this toolbox have been previously used in studies for the analysis of *in vivo* imaging data from zebrafish larvae expressing genetically encoded calcium indicators (GCaMPs)[7,9,10,25,26]. Here, we demonstrate that our integrated toolbox can be successfully applied for the analysis of the fluorescent dynamics of different calcium reporters, in different animal models and brain regions, for single-plane and multi-plane volumetric imaging, and for different imaging approaches, including large-field two-photon microscopy and single and two-photon light-sheet imaging. Moreover, we show that the module for detecting assemblies is remarkably efficient in finding biologically meaningful assemblies in datasets of whole-brain light-sheet imaging in zebrafish larvae, consisting of more than 40,000 ROIs. These examples demonstrate that the analysis framework implemented in this toolbox can effectively shed light on the neuronal interactions that underlie brain computations.

## Design and implementation

### Ethics statement

All protocols used in this study were approved by Le Comité d’Éthique pour l’Expérimentation Animale Charles Darwin (038393.03).

### Overview

Analytical demands vary greatly depending on the scientific questions and the nature of the datasets. Therefore, the pipeline is organized in flexible sub-modules that can be bypassed, replaced or used in a stand-alone manner, by allowing the user to import and integrate data and/or results from other preferred methods at different critical points along the pipeline. We provide the source Matlab code for the toolbox, along with a detailed tutorial[27] and a raw imaging dataset for a hands-on case study, to guide users in the utilization of the toolbox and demonstrate its capabilities.

**Fig 1:**
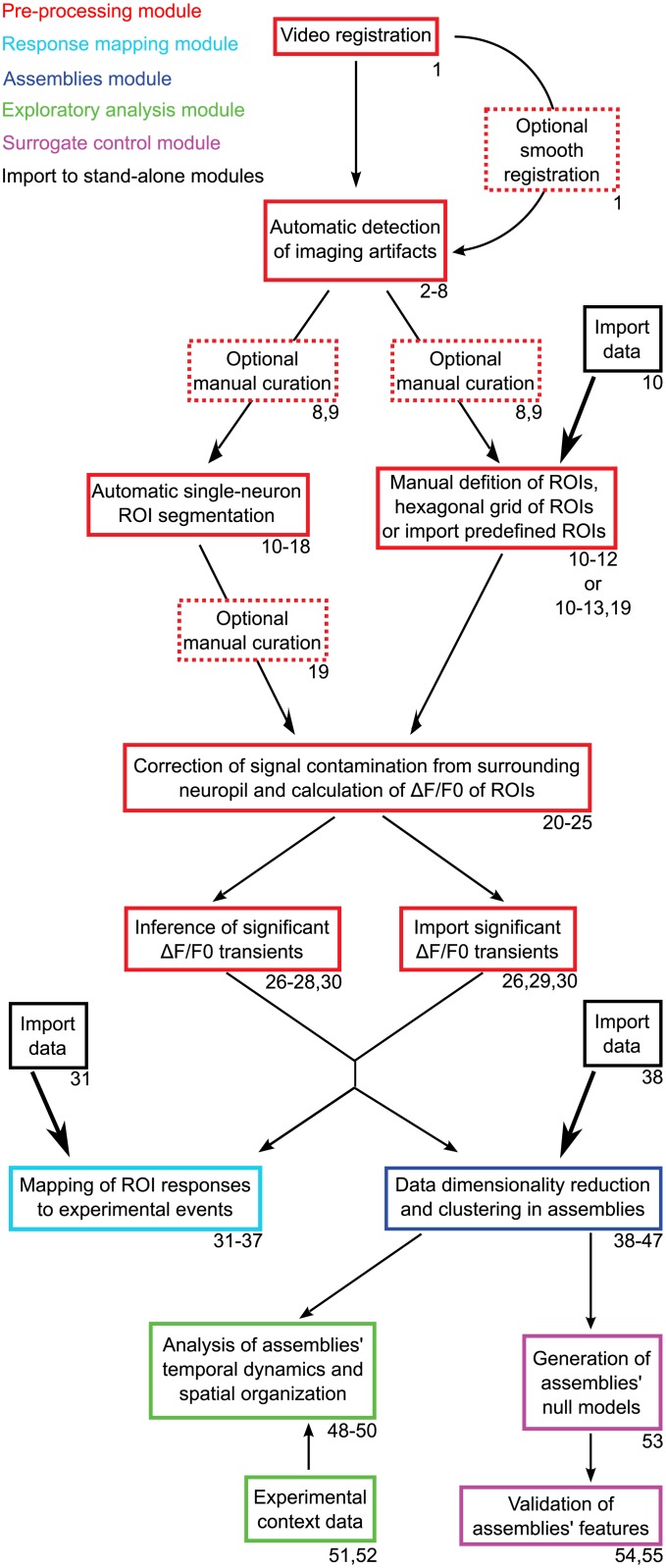
Overview of the toolbox workflow. Colored boxes indicate the processing modules (color code in top left corner). Dashed boxes correspond to optional procedures. Tutorial step numbers related to each procedure are indicated in the bottom-right corner of each box. Single-headed thin and thick arrows respectively depict the processing-pipeline flow, and the option to import pre-analyzed data from other methods into the stand-alone modules of the pipeline.

The workflow of the toolbox includes five main modules (Fig 1). The first pre-processing module contains sub-modules for: 1) smooth registration; 2) automatic detection and interactive manual curation of motion artifacts; 3) morphological single-neuron ROIs; 4) automatic detection of significant fluorescence events associated with neuronal calcium transients. The second module allows: 1) the characterization of neuronal responses (i.e., tuning curves) with respect to an experimental variable; 2) mapping the spatial topography of these responses, setting appropriate color mappings to efficiently visualize the response features across the imaged optical plane. The third module performs the detection of neuronal assemblies through different methods, including that which we recently introduced[9]. The fourth module is intended for exploratory analysis of these assemblies. It contains sub-modules for: 1) interactive exploration of the assemblies' spatio-temporal organization; 2) visualization of assemblies' activity in relation to experimental contexts and/or events (e.g., sensory stimulation, behavioral events, etc.). The fifth and final module allows for the creation of surrogate control assemblies, useful for statistical comparison against the original assemblies’ features (spatial, functional, etc). We now briefly describe the most important modules (for further details see S1 Text and the accompanying preprint[27]). A comparison to other available methods is discussed in S2 Text.

### ROI segmentation

The toolbox uses digital image processing techniques to perform morphology-based image segmentation, in order to discriminate between the ROIs that correspond to individual cells from the signal of other cells and the surrounding neuropil. Alternatively, if the imaging spatial resolution is not appropriate for single-neuron segmentation, the toolbox can automatically implement a customizable hexagonal grid of ROIs (e.g., S1A Fig), or the module's GUI can also be used to manually draw the desired ROIs, or import predefined ROIs. For simplicity, we interchangeably use the terms “ROI” and “neuron” throughout the text, except otherwise stated.

Synthetic calcium dyes label neurons rather uniformly. On the other hand, genetically encoded calcium reporters are typically expressed in the cytosol, and can be excluded from nuclei or not. Imaging neurons where the reporter is found exclusively in the cytosol results in characteristic ring-shaped fluorescent labeling. When the reporter is present in nuclei, imaging results in uniformly filled spots. Therefore, this sub-module allows the user to specify the kind of fluorescent labeling used (i.e., labeled nuclei or not), switching between algorithms tailored to detect ROIs in either of these two conditions. These algorithms are described in S1 Text. The automatic detection of ROIs is performed in a few simple and interactive steps in the GUI. First, the image of the optical plane is spatially normalized (see S1 Text for description). Then, two thresholds, cell center and neuropil border thresholds (thr_soma_ and thr_neuropil_, respectively) must be set to obtain the automatically detected ROIs. The GUI then allows the user to curate the resultant ROIs. For this, a series of ROI morphological criteria (area, fluorescent intensity, and perimeter circularity) can be applied to rapidly filter undesired ROIs. Finally, the GUI allows the user to manually add, delete or modify the ROIs.

### Correction of neuropil fluorescence contamination and calculation of ΔF/F0

The toolbox calculates the relative fluorescence variation (ΔF/F0) of each ROI, based on a few experimental parameters that the user must set. It involves performing an (optional) signal correction from neuropil fluorescence contamination, a data sanity test (see S1 Text for description), and the detection of the baseline ROI fluorescence.

Microscopy techniques are continuously improving in resolution power. However, even two-photon fluorescence microscopy has relatively limited resolution (especially axially). Thus, the neuropil fluorescence signal can substantially contaminate the somatic signal[28,29] so that F_measured_ = F_soma_ + α F_neuropil_. To overcome this, the module allows the user to set the parameter α so as to subtract a local perisomatic neuropil signal from the measured signal[11]. However, the appropriate value of α is still a matter of debate[12].

For the calculation of the ΔF/F0 of the ROIs, the user can choose to estimate F0 in two ways: i) use the average ROI fluorescence in a user-selected time window (typically, a time window immediately before a particular experimental event, e.g. sensory stimulation, animal movement, etc.); ii) use an estimation of the slow fluorescence baseline fluctuations (F_smooth_), which are unrelated to the faster calcium transients associated with neuronal activations (S1B and S1C Fig).

### Significant fluorescence transients

This sub-module automatically infers which fluorescence transients are significantly associated with neuronal activations. A few options are available, and users can choose the inference procedure that best suits their imaging experiments. Alternatively, the user can import results from alternative methods[30,31], if preferred.

The toolbox allows for the estimation of the baseline fluorescence noise scale (σ) using two options: 1) by fitting a Gaussian model to the negative ΔF/F0 fluctuations of each ROI (i.e., those below baseline, which are not related to calcium transients and hence are due to measurement noise; S1D Fig) and estimating the standard deviations of the baseline Gaussian process; 2) by estimating the standard deviations of the ROI's ΔF/F0 traces, after excluding the largest ΔF/F0 excursions (which should represent the calcium-transient peaks). Finally, we provide two options for performing the inference of significant transients. The user can choose a straightforward Static threshold method that uses a fixed fluorescence threshold for each ROI. Only those fluorescence excursions that exceed a multiple of the ROI's are considered significant. Alternatively, the user can adopt a Dynamic threshold method that exploits the estimated model of the underlying noise and applies a non-static threshold that depends on both the ROI's σ and the fluorescence decay kinetics of the calcium reporter. This method was previously described in detail[9]. Briefly, it implements a Bayesian odds ratio framework that analyzes fluorescence transitions across imaging frames. It labels as significant those transitions whose dynamics meet two conditions: i) they cannot be explained by the underlying fluorescence noise, according to a user-selected confidence threshold; ii) they are compatible with the reporter’s τ. Finally, the module automatically displays the ΔF/F0 traces and the significant transients.

### Analysis of responses

If desired, this module can be used in a stand-alone manner, independent of the previous modules of the processing pipeline, since the user can import ΔF/F0 and ROIs obtained using other procedures (see Fig 1).

The user first needs to provide timing information corresponding to the events of interest (e.g., the time of the stimulus), and the module automatically isolates, regroups and displays the event-locked single-trial and average calcium events. Significant fluorescence transients will be highlighted, allowing for the evaluation of ROI activations at the single-trial level. To plot the spatial topography of ROI responses, the module uses a hue-saturation-value (HSV) colormap, where hue represents the variable value v_peak_ that corresponds to the tuning curve peak (the ROI “preferred” variable value), saturation depicts the tuning width around v_peak_ (the ROI specificity for v_peak_), and value represents the actual ΔF/F0 value at v_peak_ (ΔF/F0_peak_; the ROI response amplitude). To make intuitive sense of the HSV color code, there must be a continuous parametric relation between the user-provided variable being mapped and the experimental event (e.g., the frequency of an auditory stimulus when mapping tonotopicity, the position of a visual stimulus when mapping retinotopicity, etc.). In some cases, the distribution of ROI response intensities may be highly skewed (a few very strong responses dominating a set of weaker ones). This can hinder the appropriate visualization of the data, if the full range of responses is represented without any color rescaling procedure. Therefore, the module allows clipping (to flatten signals that exceed a threshold) and offset of the saturation and value, improving data interpretability. Moreover, the user can choose to inversely relate ΔF/F0_peak_ to a transparency channel, which smooths out the weak and noisier responses, highlighting the most significant ones.

### Assemblies

This module can also be used as a stand-alone module independent of the previous processing pipeline (see Fig 1). To define the assemblies, the module can detect correlated ROIs by taking into account the unfiltered ΔF/F0 traces or by focusing only on the significant ΔF/F0 transients. Furthermore, it can be used to analyze single-plane or multi-plane volumetric imaging experiments.

This module applies three different clustering algorithms: i) PCA-promax clustering; ii) k-means clustering; iii) hierarchical clustering. Both ii and iii are standards and widely used clustering methods[32,33], and i was characterized and described in detail in Romano *et al*. 2015. Here we give a brief outline of the PCA-promax approach (for a longer description and discussion about the available methods for the detection of neuronal assemblies see S2 Text).

The PCA-promax implements a fully automated method that searches for ROIs with correlated calcium dynamics. Since a given ROI could belong to more than one functional group (assemblies), the PCA-promax is tailored to define non-exclusive ROI assemblies (i.e., it allows for potential overlap between the detected clusters), in contrast to classic *k-means* and hierarchical clustering. The procedure relies on previously proposed techniques[23,34]. Briefly, it consists of two processing steps. First, it z-scores the activity of each ROI and reduces the dimensionality of the complete z-scored dataset of ROI activities through principal component analysis (PCA). The initial z-scoring homogenizes the variance across ROIs, allowing PCA to reveal the global structure of ROI activity covariance. To define the assemblies, it then uses a second algorithm to partition this space of reduced dimensionality, by means of non-orthogonal factor rotation, promax[35]. This latter step extends the simpler PCA-clustering method[34] to non-exclusive assemblies.

Dataset dimensionality reduction is obtained by only keeping principal components (PCs) with eigenvalues greater than λ_max_, a theoretical lower bound to the eigenvalues of informative PCs given by the Marčenko–Pastur distribution[34,36] (Eq 1)

$$\lambda ={\left(\text{1}+\sqrt{N/T}\right)}^{\text{2}}+N^{2/3}$$ (1)

where N and T are the number of ROIs and imaging frames, respectively.

Since we are looking for non-exclusive assemblies, we relax the PCA orthogonality condition[23]. Therefore, to delineate the assemblies, the algorithm works on a space of obliquely rotated components (promax), that sparsely concentrates the PC loadings along non-orthogonal rotated PCs. Hence, after standardizing the loadings on rotated PCs by means of a z-score, a given ROI is included in a particular assembly if its z-scored loading on that rotated PC exceeds a threshold value. This threshold is easily estimated as the first minima in the distribution of z-scored maximal ROI loadings (defined by the user). The algorithm then merges clusters determined by two rotated unitary PCs if the dot product of the latter exceeds 0.6 (i.e., clusters with highly similar neuronal compositions). As a final constraint, only significantly correlated and synchronous clusters are kept (*p* < 0.05, compared to shuffled assemblies).

Finally, to assess statistical significance of the assemblies' features, the toolbox allows comparing the obtained assemblies against a set of surrogate control assemblies. For this, it allows pooling user-defined neuron features into artificial surrogate assemblies (e.g., the average neuronal activation frequencies, the neuronal tuning curves, neuronal phenotypes, pair-wise activity correlations, pair-wise topographical distances, etc.), and compare it to the pooling of the original assemblies. Certainly, the choice of control datasets that serve as null models will depend on the particular scientific question being statistically tested. We focused on two particular kinds of control datasets. We preserve the imaged fluorescence dynamics of all ROIs, and regroup ROIs according to: i) surrogate shuffled assemblies (where the ROIs of any given original assembly are randomized); ii) surrogate assemblies that preserve the original assemblies' spatial features (i.e., conserving the distribution of the relative pair-wise physical distances between ROIs in the original assemblies).

## Results

Given the large scope of the pipeline, here we focus on the typically obtained results of the most relevant processing stages. The processing of all datasets was performed according to the tutorial provided.

As described in the tutorial, the initial data pre-processing consisted in registering in-plane x-y drifts of the imaged optical section, followed by the automatic detection of movement-related imaging artifacts. Then, the toolbox was used to automatic detection and interactive manual curation of morphological single-neuron ROIs, as explained next.

### Segmentation in single-neuron ROIs

As expected, the success of this module depends on the image's spatial resolution, the anatomy of the imaged region, and the cellular labeling by the fluorescent reporter. Here, we demonstrate that it can successfully process remarkably different datasets.

**Fig 2:**
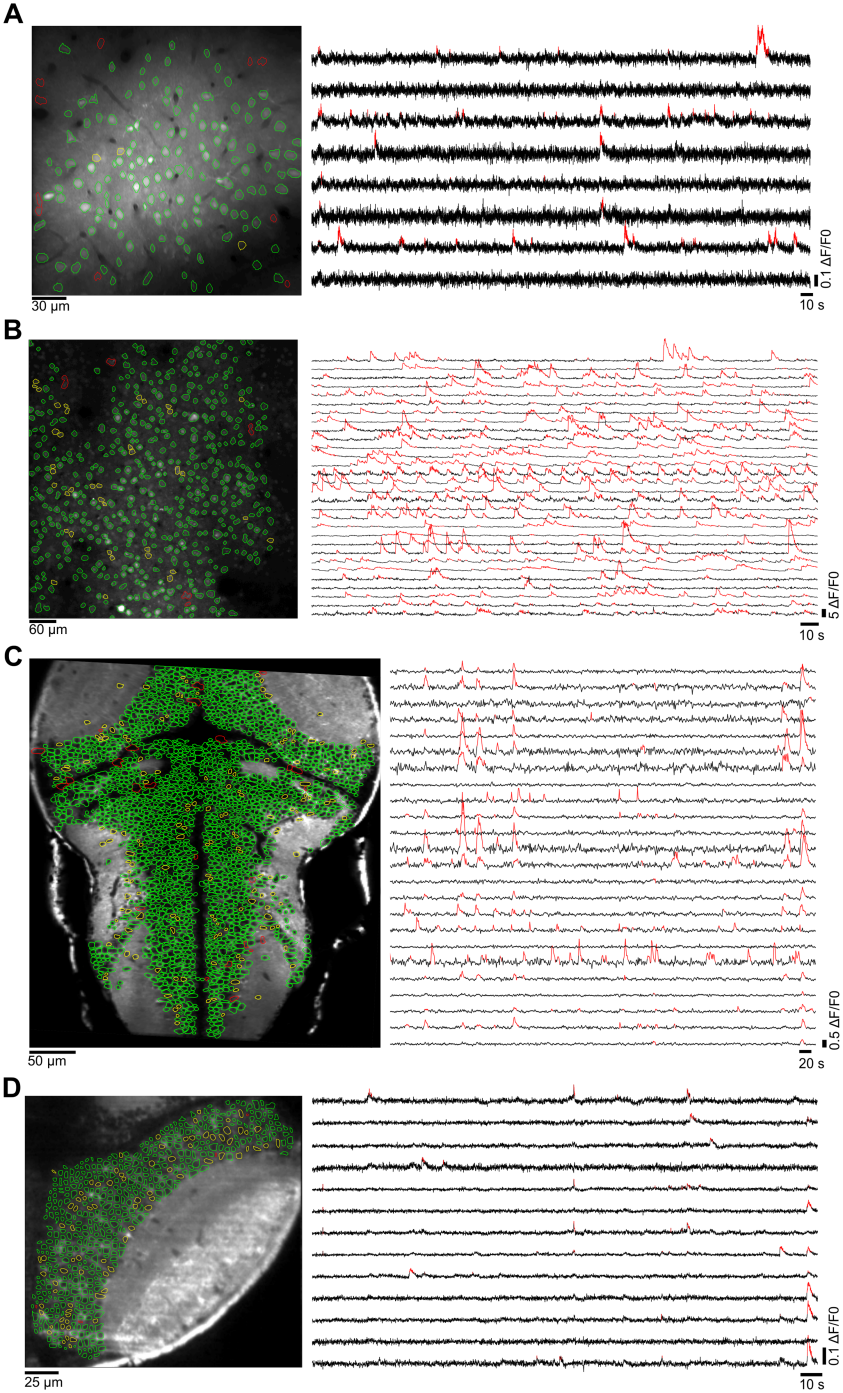
Detection of ROIs corresponding to single neurons. Four examples from different animal models, brain regions, imaging techniques and calcium indicators. Left: imaged optical planes. Perimeters of correct and incorrect automatically detected ROIs are shown in green and red, respectively. Yellow perimeters, detected ROIs that had to be manually curated, or undetected but manually drawn. Right: representative Δ*F/F0* traces (black) and significant fluorescent transients detected (red). (**A**) Mouse primary visual cortex bolus injected with OGB-1 AM (two-photon imaging, 256x256 pixels, 30 Hz sampling rate). Data from Scholl *et al*[37]. Segmentation was performed with the “labeled nuclei” and “bigger ROI” options, and we set parameters “local contrast” to ~20 for spatial normalization of the image, thr_neuropil_ and thr_soma_ to ~0.45 and ~0.095, respectively, and “minimal ROI area” to 18 pixels. (**B**) Mouse somatosensory cortex, where nuclei of excitatory neurons are transgenically labeled with mCherry (two-photon imaging, 256x256 pixels, 7 Hz sampling rate). Calcium dynamics monitored with GCaMP6s. Data from Peron *et al*[11,38]. Parameters used: “labeled nuclei” and “bigger ROI” options, “local contrast” ~43, thr_neuropil_ ~0.15, thr_soma_ ~0.08, and “minimal ROI area” set to 15 pixels. (**C**) Transgenic zebrafish larva pan-neuronally expressing GCaMP3 (two-photon imaging, 512x256 pixels, 1 Hz sampling rate). Parameters used: “unlabeled nuclei” and “smaller ROI options”, “local contrast” ~10, thr_neuropil_ ~0.15, thr_soma_ ~0.01, “minimal” and “maximal ROI areas” set to 7 and 40 pixels, and “minimal” and “maximal circularity” to ~0.48 and ~1.7. (**D**) Right hemisphere of the optic tectum of a transgenic zebrafish larva pan-neuronally expressing GCaMP5 (single-photon light-sheet imaging, 232x242 pixels, 100 Hz sampling rate). Parameters used: “unlabeled nuclei” and “smaller ROI options”, “local contrast” ~20, thr_neuropil_ ~0.15, thr_soma_ ~0.01, “minimal ROI areas” set to 8 pixels.

We provide 5 examples of single-neuron ROI detection performance obtained with two-photon imaging: i) for an injected synthetic dye that labels the entire volume of the neurons (OGB-1 AM) in mouse visual cortex[37] (Fig 2A; dataset pvc-10 from crcns.org); ii) for mCherry-labeled nuclei in a GCaMP6s imaging experiment obtained by viral injections in the mouse somatosensory cortex[11,38] (Fig 2B; dataset ssc-1 from crcns.org); iii) for large-field imaging of nuclei-excluded GCaMP3 in transgenic zebrafish larvae (Fig 2C); iv) for a preparation similar to iii but imaging a smaller field (the provided case study); and v) an example of single-neuron ROI detection in single-photon high-acquisition rate (100 Hz) light-sheet imaging of a transgenic zebrafish expressing GCaMP5 (see Fig 2D). These examples illustrate the algorithm's performance in settings that differ markedly in the density of neuronal labeling (examples iv and i being the most and less dense, respectively), and with imaging techniques of different spatial resolution and acquisition rates (two-photon laser scanning and single-photon light-sheet microscopies).

For both mouse cortical datasets (Fig 2A and 2B) it took less than 5 mins to obtain 149 and 505 ROIs, respectively. For the large-field dataset in zebrafish (2178 ROIs), it took ~10 mins (Fig 2C). Finally, for the light-sheet imaging dataset we obtained 438 ROIs in less than 10 mins (Fig 2D). Typically, we obtained a >85–90% success rate in automatically detecting ROIs corresponding to single neurons, depending on imaging quality. For the present examples, after setting the parameters for ROI segmentation and filtering, we had to curate 8% of the ROIs for the case study, 9% for both mouse cortical datasets (Fig 2A and 2B), while for the large-field zebrafish dataset, 8% of the ROIs were curated (Fig 2C). For the single-plane single-photon light-sheet imaging dataset shown in Fig 2D, we had to curate 15% of the ROIs, due to the lower spatial resolution of this technique. Since simultaneous multi-plane light-sheet imaging does not guarantee single-neuron resolution, we used an array of hexagonal ROIs.

### Detection of fluorescence transients related to neuronal activity

**Fig 3:**
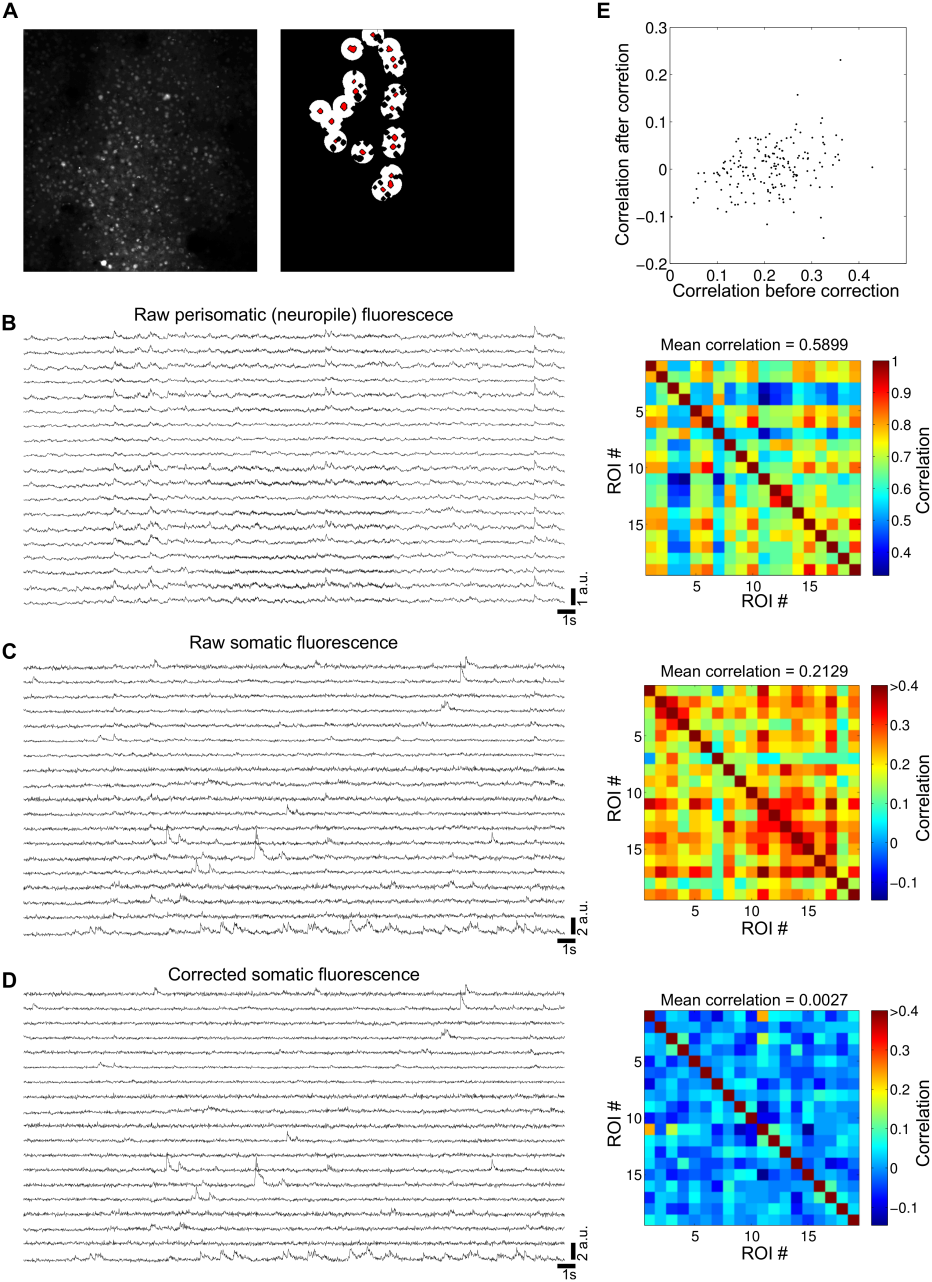
Correction of neuropil fluorescence contamination. (**A**) Left: optical plane imaged with two-photon microscopy of the mouse somatosensory cortex (same data as Fig 2B). Right: examples of the detected ROIs (red) and their circular perisomatic masks used to calculate the local neuropil signals (white). Perisomatic masks are overlapping, but each ROI is associated with a single circular mask. Black holes inside the perisomatic masks are other detected ROIs not included in the local neuropil signal calculation. (**B**) Left: raw fluorescence traces obtained with the perisomatic masks shown in **A** (neuropil signal). Right: pair-wise correlation matrix for the signals shown in the left. Note the high temporal correlation across the traces. (**C**) Same as **B**, for the raw fluorescence traces of the ROIs shown in **A** (somata). (**D**) Same as **C**, for the corrected ROI fluorescence traces, obtained by subtracting the traces shown in **B** from the corresponding traces shown in **C**, with α = 0.9. Note the reduction in the temporal correlations, compared to those found in **C**, despite the small changes of the individual fluorescence traces. (**E**) Relationship between the pair-wise correlations shown in **C** and **D**.

To correct from neuropil fluorescence contamination, for each ROI, the corresponding local neuropil signal is automatically obtained from a circular area with a 20 μm radius that surrounds the ROI in question and excludes all other ROIs (Fig 3A). Neuropil subtraction is particularly important when imaging loosely scattered neurons surrounded by neuropil (e.g., Fig 2A and 2B). Indeed, signal contamination from the neuropil can notably affect the measurement of the correlations between the neuronal fluorescence time courses (Fig 3B–3E).

The inference of significant ΔF/F0 transients can be applied to a variety of imaging conditions (Fig 2). To infer the statistical significance of fluorescence transients, the algorithm implemented in the toolbox considers that any event in the fluorescence time series data belongs to either a neuronal activity process or to an underlying noisy baseline. Thus, the toolbox first performs a key step for this inference, which is the estimation of the ROIs' fluorescence noise levels (i.e. the baseline fluorescence noise scale σ). With this estimate, the toolbox can detect significant transients, by imposing a static fluorescence threshold based on the ROI’s σ, or, alternatively, using a simple dynamic threshold that analyzes fluorescence transitions, depending on both the ROI's σ and the biophysics of the imaged calcium indicator. The latter method imposes stronger constraints to the data, but is more robust against false positives. Therefore, it is particularly useful for imaging conditions associated with lower signal-to-noise ratios (SNR; e.g., when imaging large fields with low-intensity excitation light).

For the mouse cortical data (Fig 2A and 2B), the ROIs’ baseline noise σ was estimated with the Gaussian model, and the significance of fluorescence transients was inferred with the Dynamic threshold method with the appropriate τ of the calcium reporters. For the light-sheet imaging datasets in Figs 2D and 8, σ was also estimated with the Gaussian model, but due to the large size of the datasets, significant transients were obtained with the faster Static threshold method.

**Fig 4:**
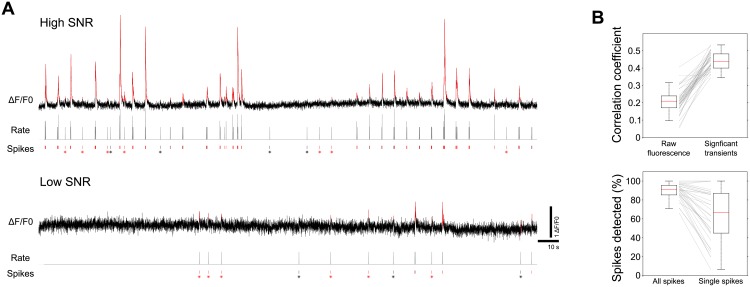
Toolbox performance in inferring neuronal activity from calcium imaging data. (**A**) Two examples with different signal-to-noise ratios (SNRs) from different neurons in the cai-1 ground-truth dataset[28,39]. For each example, we show: top, GCaMP6f ΔF/F0 traces (black) and the significant fluorescent transients detected by the module (red); bottom, ticks representing the simultaneously recorded spikes (those associated with a significant calcium event are highlighted in red; asterisks mark single spikes); middle, spiking rate of the neuron calculated by temporally convolving spikes with a Gaussian filter of σ = 20 ms. Imaging was performed at 60 Hz. A spike was considered as associated with a significant calcium event if it was followed by an event of significant fluorescence within 40 ms (GCaMP6f rise time τ_peak_ = 45± 4 ms, for 1 spike[28]). Significant transients were calculated with default toolbox parameters in Dynamic threshold mode, with a GCaMP6f τ_decay_ = 250 ms[28]. (**B**) Boxplot summary of performance for all recordings (n = 37). Top: coefficients obtained when correlating the spiking rates with the raw fluorescence traces or with the ΔF/F0 traces of significant transients (where non-significant fluctuations were set to 0). Bottom: Percentage of “detected” spikes (i.e., those associated with a significant calcium event), for all recorded spikes or for single spikes only.

We quantified the toolbox performance in detecting neuronal activity from imaging data. For this, we applied the dynamic threshold method to a dataset[28,39] consisting of simultaneous imaging and loose-seal cell-attached recordings in GCaMP6f expressing neurons (37 recordings from 11 different neurons; Fig 4; dataset cai-1 from crcns.org). Using this ground-truth data, we observed that the significant fluorescence transients inferred by the algorithm correlated well with the firing rate of the neurons (*r* = 0.44±5; top panel in Fig 4B). This is comparable to the best performance obtained by more complex spike-inference algorithms applied to the slower GCaMP6s signal (compared to that of GCaMP6f), albeit imaged in more challenging conditions[40]. Moreover, 90±9% of all the recorded spikes, and 63±28% of the single spikes (spikes separated to other spikes by at least 1 s), were related to a significant calcium transient (bottom panel in Fig 4B). Overall, this suggests that, when imaging calcium reporters with fast kinetics (e.g., GCaMP6f), the toolbox can confidently detect in an unsupervised manner significant fluorescence transients related to action potentials, without the need for training data or more complex and supervised inference algorithms.

### Analysis of neuronal responses

Several studies aim to relate neuronal activity to experimental events (e.g., sensory stimulation, behavioral response, etc.). Indeed, imaging is particularly suited for revealing the spatial distribution of neuronal responses (i.e., topographic sensory/behavioral functional maps), which may be crucial to understand brain coding strategies. The toolbox allows associating ROI fluorescence responses with a given experimental variable (provided by the user), and visually displays them on the imaged optical plane to assess their topographical organization.

**Fig 5:**
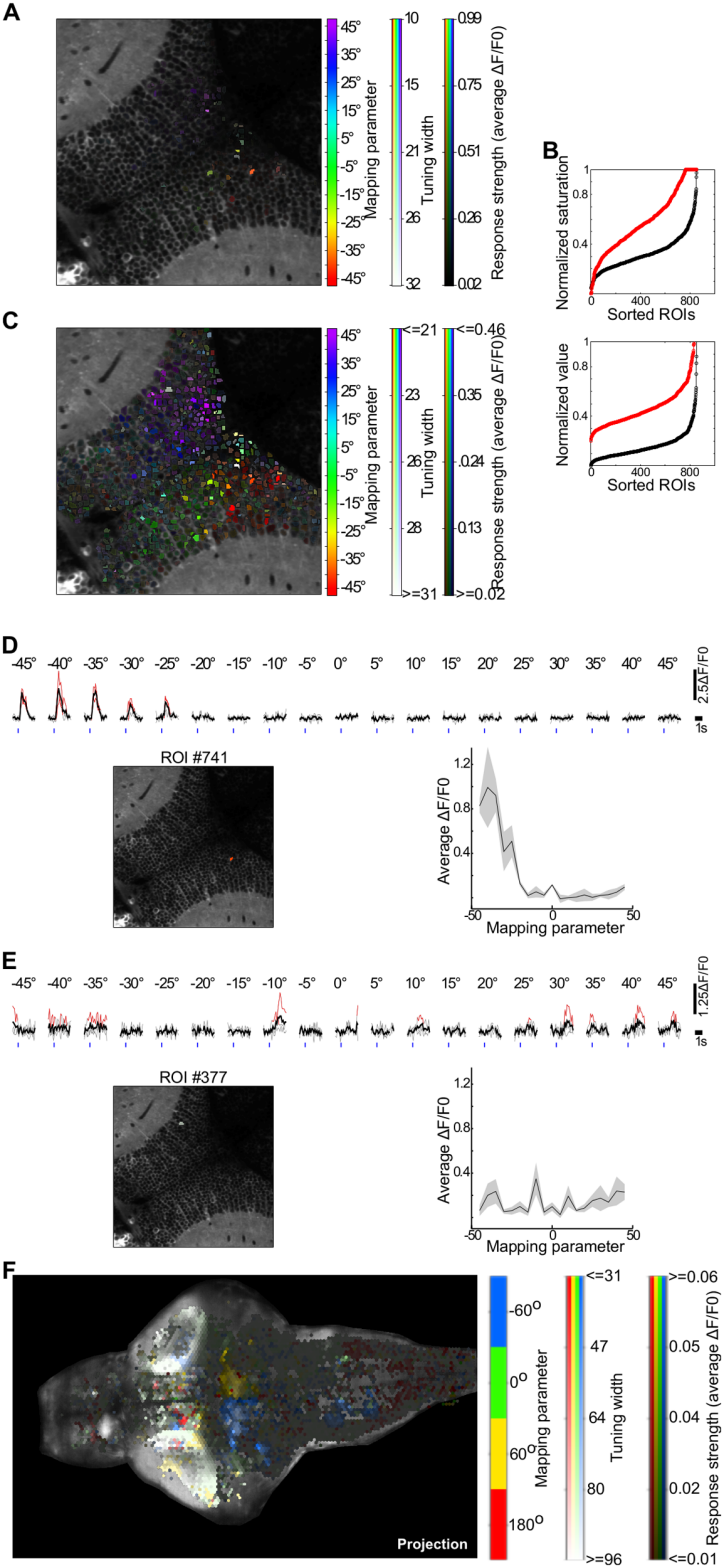
User interface screenshots of the calculated neuronal responses associated with experimental events. (**A-E**) Responses to visual stimulations with light spots at different azimuth angles of the larva’s visual field (two-photon imaging of a GCaMP3-expressing zebrafish larva). (**A**) ROIs are colored with an HSV color code representing their preferred azimuth angle (Peak mapping parameter; hue), azimuth selectivity (Tuning width; saturation) and average response at preferred azimuth (Response strength; value). Due to the skewed distribution of the responses, only a few responsive ROIs can be visualized. (**B**) Offsetting and clipping of the saturation and value channels to improve visualization. Black, original values used in **A**; red, rescaled values used in **C**. (**C**) Same data shown in **A**, but with the rescaled channel ranges. After this step, the retinotopic organization of the optic tectum becomes evident. (**D** and **E**) Screenshots of the responses of two ROIs selected by clicking on Select ROI in **c**. Average (black) and single-trial (gray) ΔF/F0 responses are organized according to the stimulus values, where significant trial responses are shown in red. Bottom right, tuning curves of the ROIs. Black, mean response; gray patch, standard error. Note how the responsive but less selective ROI in **e** is shown with a more whitish color code (low saturation). (**F**) Responses to visual stimulations with gratings moving in angular directions, monitored by volumetric light-sheet single-photon imaging of a GCaMP5-expressing zebrafish larva. Responses are displayed in HSV color code over the maximal intensity projection of all imaged optical sections. Note that, while the tectal neuropil indiscriminately responds to all directions (whitish ROIs), a pair of bilaterally symmetric group of ROIs in the hindbrain responds selectively to either 60° or -60° (see S1 Video for volumetric distribution of responses).

To illustrate the capabilities of the response-analysis module, we present the results obtained with two different datasets. The first one is the case study included in the toolbox tutorial (Fig 5A–5E). For this case study, GCaMP3 was imaged to monitor the responses of the zebrafish optic tectum to visual stimulation consisting of 3 trials of 4° light spots presented at different azimuths of the visual field (-45° to 45° in 5° steps, 0° represents directly in front of the larva). Event-locked single-trial and trial-averaged ROI ΔF/F0 responses are automatically displayed (see Fig 5D and 5E), allowing the interactive exploration of any ROI of interest. Moreover, the information in these trial responses is also summarized by calculating the “tuning curves” of each ROI. For this purpose, the module plots the trial-averaged ROI ΔF/F0 responses as a function of the experimental variable (see bottom right panels in Fig 5D and 5E). Finally, these ROI tuning curves are color-coded and superimposed on the imaged optical plane, using the hue-saturation-value (HSV) colormap described in Design and Implementation (see Fig 5C). We observe that neighboring visual-field positions are mapped on neighboring positions in the optic tectum, reflecting the tectum’s functional retinotopic map[41,42] (Fig 5C). To improve the visualization of the topography of responses, we used the GUI (Fig 5B) to clip and offset the saturation and value channels (compare Fig 5A and 5C).

The second dataset used was obtained through multi-plane light-sheet imaging, where the activity of >40,000 simultaneously monitored ROIs were sampled at 2.1 Hz in a GCaMP5-expressing transgenic zebrafish larva. Since fast volumetric light-sheet imaging does not guarantee single-neuron resolution, we used arrays of hexagonal ROIs covering the totality of each of the 40 optical planes imaged (separated by steps of 5 μm). Every 20 min, the larva was visually stimulated using whole-field gratings capable of inducing optomotor response (OMR; in which larva turn and swim to follow the whole-field motion). The gratings moved in different directions (-60°, 0°, 60° and 180°, where the angle indicates the difference between the larva's heading direction and the direction of the moving grating; 24 trials of 10 s per moving direction). Using the response-analysis module, we automatically computed the spatial distribution of ROI responses, observing a bilaterally symmetric group of ROIs located in the hindbrain, tuned to stimuli that elicit OMR in opposite directions (gratings moving at 60° and -60°), revealing brain regions whose activations are related to the sensorimotor transformation (Fig 5F and S1 Video).

### Detection of neuronal assemblies

The analysis of responses associated with particular experimental events, as those illustrated in the previous section, can provide insights into neuronal functioning. This is a kind of supervised analysis that requires explicit timing information in order to calculate the event-locked ROI responses. Nevertheless, the analysis of the activity dynamics of neuronal populations can also reveal organizational principles in an automatic and unsupervised manner. Here, we focus on the unsupervised detection of neuronal assemblies (i.e., clusters of ROIs with similar activity dynamics)

**Fig 6:**
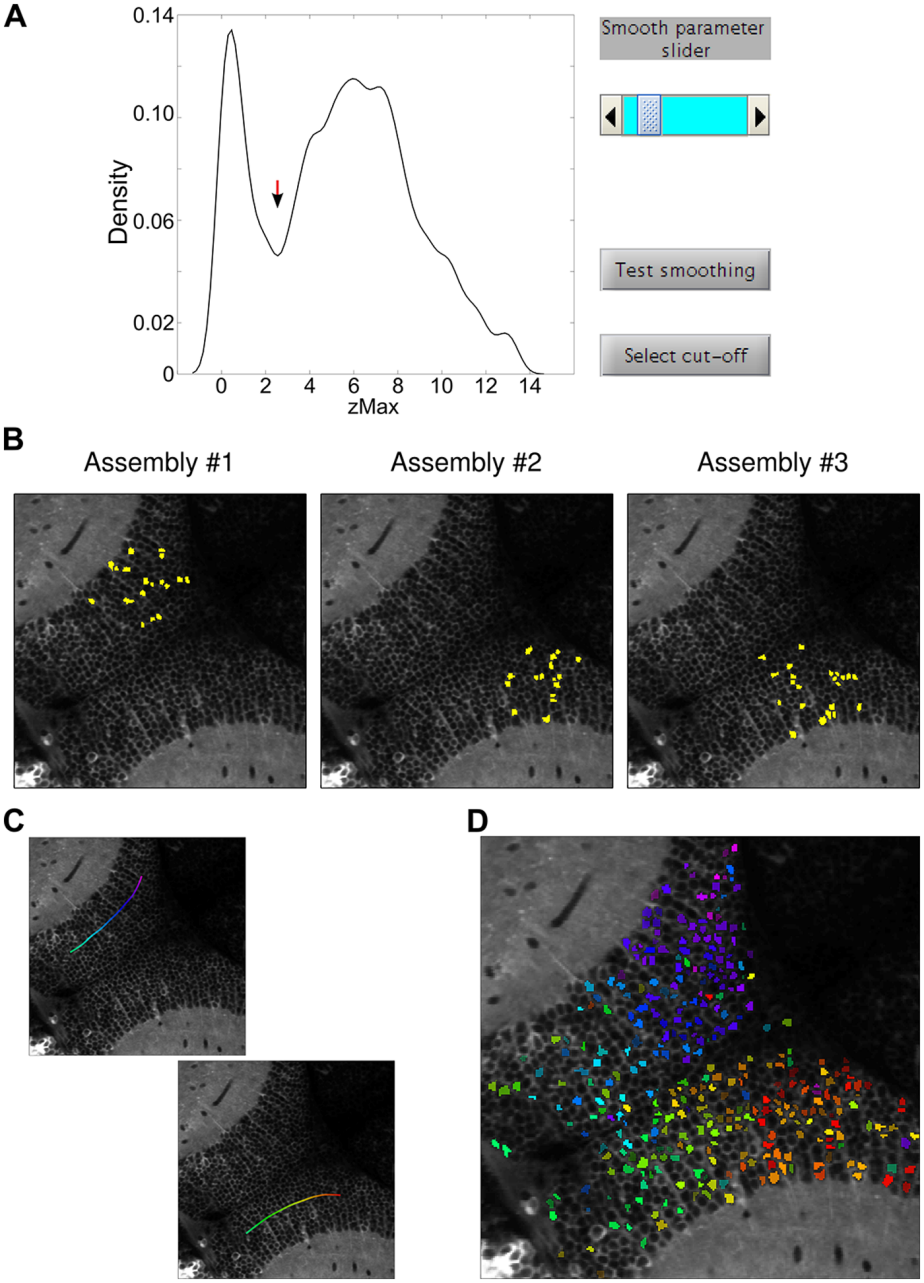
Detection of neuronal assemblies for the case study. (**A**) Screenshot for the selection of the zMax threshold to determine the neuronal composition of the assemblies. After setting the smooth parameter with the slider (top-right), the threshold is chosen with a mouse click on the graph of the density distribution (red arrow). (**B**) Screenshot showing the topography of 3 representative assemblies (out of 42). ROIs that belong to each assembly are labeled in yellow. (**C**) Screenshot of two user-defined anatomical axes (see tutorial) over which assemblies will be spatially organized. Each curve is automatically colored so that the combined curves reproduce the hue gradient used in Fig 5A and 5C. The chosen curves span the rostro-caudal retinotopic axis of each tectal hemisphere. (**D**) Screenshot of the figure obtained displaying the spatial organization of the assemblies along the selected axes. Assemblies' ROIs are colored according to the defined axis (i.e., the position of the assemblies’ spatial centroid with respect to the defined axis). The comparison with Fig 5C confirms that assemblies reproduce the tectal retinotopic functional map.

For the detection of neuronal assemblies in the case study of the tutorial, we used the PCA-promax method. We chose to manually select the single input parameter required by the program, the zMax threshold (Fig 6A). Examples of the spatial topographies of the detected assemblies are shown in Fig 6B, which reveal a majority of spatially compact neuronal clusters. Remarkably, displaying these assemblies color-coded according to their spatial centroid position (Fig 6C and 6D), clearly recapitulates the functional retinotopic map of the optic tectum (Fig 5C). As explained before, this dataset consists of tectal light-spot-induced neuronal responses. Thus, this demonstrates that the clustering procedure is capable of exposing the functionally relevant spatial organization of tectal responses, without any information on the timing of the stimulation.

**Fig 7:**
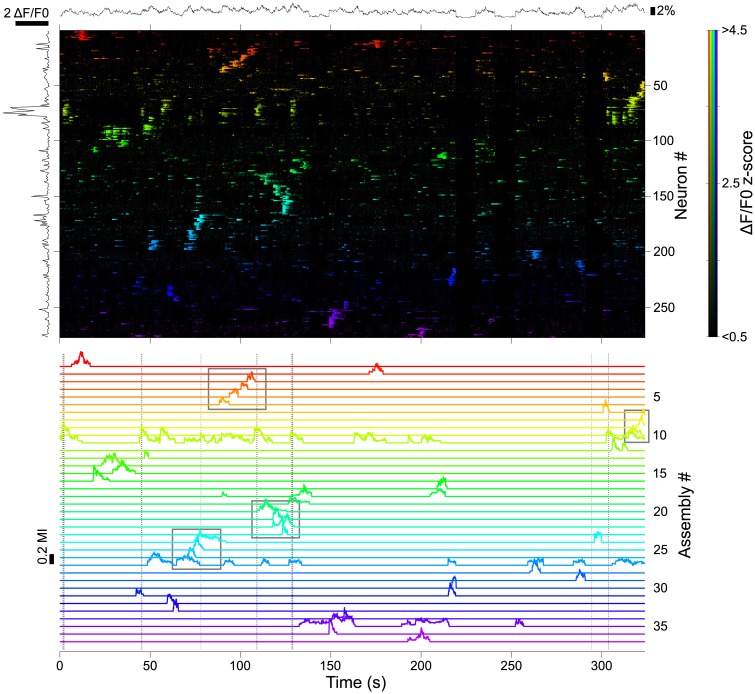
Detection of neuronal assemblies in the mouse barrel cortex. Top: raster plot of the z-scored ΔF/F0 of 277 neurons distributed in 37 assemblies, from the total imaged population of 1025 neurons. Neurons are sorted and color-coded according to the assembly to which they belong to (color bar on the right). Black trace on top, fluctuations of the number of active neurons in the total imaged population; Black trace on the left, average neuronal responses to a whisker-object contact. Bottom: activation dynamics of the detected assemblies, color-coded as in the raster plot. Vertical dotted lines indicate moments of whisker-object contact. Note that activations of assembly #11 are associated with contacts, and episodes of activity sequentially progressing through assemblies (marked by gray boxes).

We applied the same PCA-promax procedure on the mouse somatosensory cortex GCaMP6s-imaging dataset (the same of Fig 2B). In this experiment, the superficial barrel cortex was imaged while the mouse performed a single-whisker object localization task. Activity fluctuations along the experiment were limited to <5% of total neuronal population imaged (1025 neurons), however, clustering neurons according to their activities revealed the coordinated activation of several sets of neurons Fig 7). Interestingly, one of these neuronal assemblies (assembly #11) consisted of a group of correlated neurons that was responsive to the mouse contacting the whisker with the object. Apart from detecting this group of functionally related neurons, the clustering algorithm also exposed the ongoing concerted activity of other assemblies along the task, that may convey additional contextual or task-related signals.

**Fig 8:**
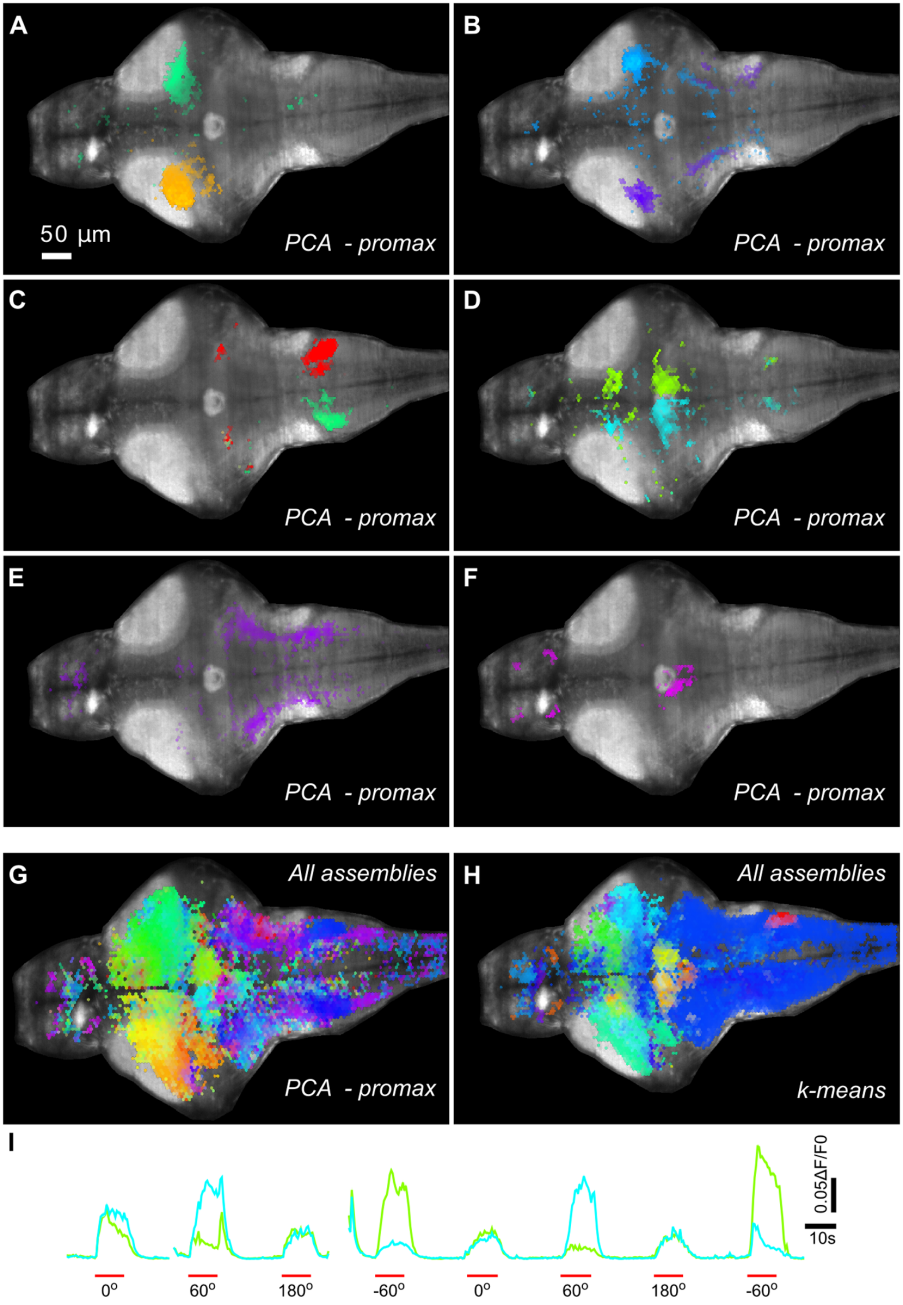
Neuronal assemblies obtained using whole-brain light-sheet imaging in zebrafish reveals the spatial structure of brain activity. A GCaMP5-expressing larva was volumetrically scanned with fast multi-plane single-photon light-sheet microscopy. Due to the lack of single-neuron resolution in this imaging dataset, a grid of 9 μm-diameter hexagons was imposed over each imaged optical plane. (**A-F**) Some of the ROI assemblies found with the PCA-promax method, displayed over the maximal intensity projection of all imaged optical sections (for individual optical sections see S2 Video). (**A-D**) Pairs of symmetric unilateral assemblies. (**E-F**) Single bilaterally symmetric assemblies. (**G**) All ROI assemblies found with the PCA-promax approach, displayed over the maximal intensity projection of all imaged optical sections (for individual optical sections see S3 Video). Assemblies are colored according to the similarity of their activity dynamics (the more temporally correlated, the more similar the color of the assemblies). This color code was also used in the previous panels (except for the assembly pair shown in **c**, which was colored differently to facilitate visualization). (**H**) Same as **G**, but for assemblies found using k-means, color-coded according to the similarity of their activity dynamics. To allow for a comparison between **G** and **H**, the dimensionality of the dataset was reduced through PCA before clustering (otherwise, clustering did not converge). Note how this clustering reveals assemblies whose spatial organization is roughly consistent with those shown in **G**, but exposes a much less biologically relevant fine structure. (**I**) Single-trial activation dynamics of the assemblies shown in **D** during one of the blocks of visual stimulation. Periods of moving-grating stimulation are labeled in red, angles indicate the grating's moving directions. Note that both assemblies are activated by this OMR-inducing stimulation, with the bluish and greenish assemblies preferring 60° and -60°, respectively.

This kind of analysis can also be performed in large-scale activity data. In Fig 8 and S2 and S3 Videos, we illustrate the result of using the PCA-promax method to detect assemblies in a brain-wide light-sheet imaging dataset, and compare it to the k-means clustering. The algorithm revealed several bilaterally symmetric assemblies and unilateral assemblies with their corresponding contralateral counterparts. In particular, we observed a symmetric pair of assemblies distributed over the pre-tectum and the anterior and caudal hindbrain (Fig 8D and S2 Video), whose activities were tightly related to experimental events (OMR-inducing visual stimulation; Fig 8I), stressing their relevance. In agreement with recent findings[43,44], this particular assembly was also apparent when averaging ROI responses during OMR stimulation (Fig 5F and S1 Video), but the average-response map was much noisier and less anatomically defined than the assemblies found through activity clustering. Moreover, we also found topographically precise clusters that were missed by averaging ROI responses to visual stimulation, like a symmetric pair of assemblies comprising the midbrain torus semicircularis, along with its octavolateral (lateral line and acoustic) afferences from the hindbrain (Fig 8B).

These examples illustrate the capability of PCA-promax clustering to expose the biologically relevant spatio-temporal organization of high-dimensional datasets, going beyond the analysis of trial-averaged responses by allowing the study of single-trial population dynamics.

**Fig 9:**
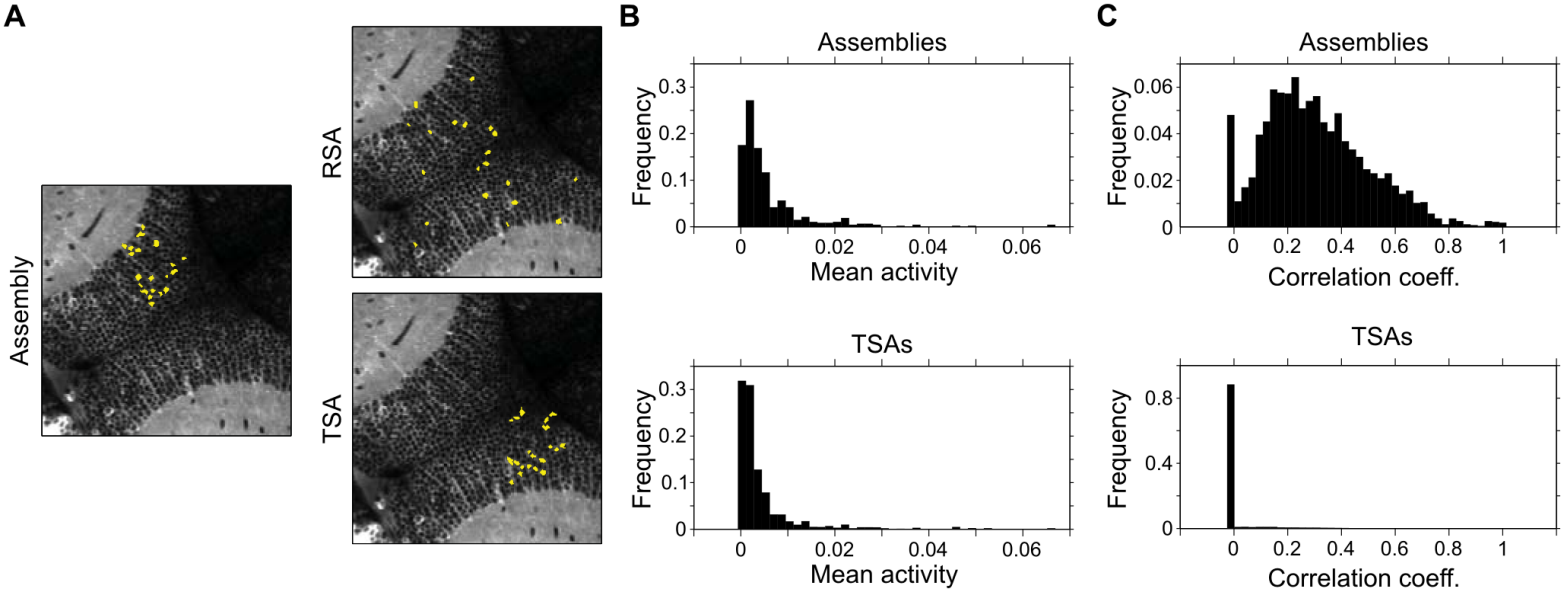
Comparison of specific features of the assemblies with those of surrogate controls. (**A**) Spatial layouts of a given assembly detected in the optic tectum case study, and of one example of a Random surrogate assembly (RSA, where an equal number of ROIs are randomly placed) and a Topographical surrogate assembly (TSA, where ROIs are placed preserving the inter-ROI distances of the original assembly). (**B**) The normalized frequency histogram of average ROI activity levels (mean significant ΔF/F0 per imaging frame) obtained for ROIs included in assemblies (top) and those included in TSAs. (**C**) Same as **B**, but for the ROI activity correlations (pair-wise Pearson correlation coefficient).

Finally, when validating results obtained from large multidimensional datasets, the use of appropriate control surrogate datasets is critical. As an example, since neighboring neurons tend to be more highly correlated, we asked if the spatially compact assemblies of the optic tectum case study were the result of simply grouping nearby neurons. We demonstrate that this is not the case, since we found that compared to Topographical surrogate assemblies, the assemblies of the optic tectum do not show any particular trend in the average activity level of their component ROIs (Fig 9B), but they do significantly group correlated ROIs (Fig 9C). This example allows the user to further validate the clustering results, which effectively groups correlated ROIs, without showing a bias for the most active ones (a common concern in clustering procedures).

## Availability and future directions

The toolbox is an open-source project freely available to the community. It was developed under Matlab and verified to run properly through a variety of versions, from R2010a up to 2015b. It requires the Matlab’s Curve Fitting, Image Processing, Statistics and Machine Learning, and Parallel Computing toolboxes. The source code can be downloaded, together with a detailed tutorial[27] and a case study dataset, from https://github.com/zebrain-lab/Toolbox-Romano-et-al. Data analyzed here includes the case study included in the toolbox, and datasets downloaded from https://crcns.org/data-sets/ssc/ssc-1, https://crcns.org/data-sets/vc/pvc-10 and https://crcns.org/data-sets/methods/cai-1. The pipeline can be run on any desktop computer, but we recommend multicore computers for shorter computing times, as several algorithms allow for parallelization. For analysis of large light-sheet imaging dataset, we used a computer cluster of 328 CPUs based on an HTCondor parallelization system. Imaging videos of long experiments can result in file sizes of several gigabytes (GB), and therefore we recommend 64-bit architectures with at least 12 GB of RAM. We also recommend screen resolutions of at least 1280x800 pixels for a better experience with the GUI.

While calcium indicators have become the standard for imaging neuronal activity, genetically encoded voltage indicators (GEVIs) are a promising technology [45,46], despite important constraints on the technique[12]. In principle, the toolbox could be extended without difficulty to allow the analysis of imaging data using GEVIs. Furthermore, clustering of large multivariate datasets is a complex mathematical problem. The PCA-promax method for detecting assemblies relies on PCA, which implements linear data transformations. Thus, in the future the toolbox could be supplemented with other measures of activity similarity to better capture non-linear neuronal activity correlations, like manifold learning algorithms[47,48] and network theory[49]. Nevertheless, new mathematical techniques to address this issue are eagerly awaited, since unsupervised faithful learning of non-linear interdependencies across large neuronal populations is an extremely challenging task that remains unresolved.

## Supporting information

S1 Text Further details on the toolbox. (PDF)

S2 Text Comparison to other methods. (PDF)

S3 Text Installation instructions for the toolbox. (PDF)

S1 Fig Pre-processing fluorescence dynamics. (**A**) Red, hexagonal grid of 6 mm-diameter, over an imaged optical section of optic tectum of a zebrafish larva pan-neuronally expressing GCaMP3. The region covered by the grid is defined with a user-drawn mask. (**B**) Left, raw fluorescence of a ROI (black) and the estimated F_smooth_. Right, ΔF/F0 obtained using F_smooth_ as F0. Note how slow fluctuations are removed, producing a stable ΔF/F0. (**C**) Zoom of **A**. Note how follows slow fluorescence variations, ignoring the fast, neuronal activity related fluorescence transients. (**D**) Estimation of the baseline fluorescence noise (σ) of a ROI. Black, normalized histogram of the ROI's ΔF/F0; red, Gaussian fit to the negative fluorescence ΔF/F0.
(PDF)

S2 Fig Mapping of responses to HSV color-code. (**A**) Schema illustrating the definition of the hue, saturation and value to visually represent ROI responses in a color-code. Left, ROI tuning curve. Arrows indicate the particular hue, saturation and value of this ROI. Right, colorbars representing the range of hue, saturation and value for all the imaged ROIs. Arrows indicate the color-code parameters for the ROI tuning curve shown in the left. (**B**) Display of neuronal responses only representing preferred stimulus (i.e., the peak mapping parameter). Same as Fig 5, but disabling saturation and value channels, thus only representing preferred stimulus on the hue channel. The noisier image obtained underscores the utility of additionally representing the neuronal selectivity and response strength.
(PDF)

S1 Video Volumetric distribution of ROI responses shown in Fig 5F. The axial projection of all the ROI responses is initially displayed, followed by each one of the 40 imaged optical planes (plane depths are indicated in lower right corners).
(MP4)

S2 Video Volumetric distribution of example assemblies shown in Fig 8A–8F. Assemblies obtained with the PCA-promax algorithm. For each panel, the corresponding axial projection of the example assemblies is initially displayed, followed by their layout over each one of the 40 imaged optical planes (plane depths are indicated in lower right corners).
(MP4)

S3 Video Volumetric distribution of all the assemblies shown in Fig 8G. Assemblies obtained with the PCA-promax algorithm. The axial projection of all the assemblies found in the dataset is initially displayed, followed by each one of the 40 imaged optical planes (plane depths are indicated in lower right corners).
(MP4)
