# Supplementary material for: An integrated calcium imaging processing toolbox for the analysis of neuronal population dynamics
Source: PLoS Comput Biol. 2017 Jun 7;13(6):e1005526. doi: 10.1371/journal.pcbi.1005526 (PMC5479595; doi:10.1371/journal.pcbi.1005526)
Supplement: S1 Text — (PDF) [file pcbi.1005526.s001.pdf]

FURTHER DETAILS ON THE TOOLBOX

ROI segmentation

The steps of the ROI segmentation algorithm are the following. First, the imaged video is averaged across frames to obtain the image *avgImg*. Fluorescent labeling may be uneven in the imaged plane. Therefore, the intensity of *avgImg* must be spatially normalized to avoid biases in the segmentation. For this, we first filter *avgImg* with either a 10% or a 90% order-statistic filter (with a running square window), obtaining $avgImg_{10}$ and $avgImg_{90}$, respectively. We then calculate the spatially normalized image $avgImg_{Norm}$ as $avgImg_{Norm} = (avgImg - avgImg_{10}) / (avgImg_{90} - avgImg_{10})$. The user can set the size of the square running window so that $avgImg_{Norm}$ clearly shows the fluorescent-labeled cells with an intensity that does not vary significantly across the imaged plane. If cell nuclei are unlabeled, $avgImg_{Norm}$ is converted to its complement (i.e., its negative), converting the unlabeled nuclei into bright spots. The user then has to interactively set two threshold parameters, thr_{Soma} and $thr_{Neuropil}$, that will result in binary masks that identify cell somata and the regions that surround them (i.e., the neuropil) , $Mask_{Soma}$ and $Mask_{Neuropil}$, respectively (see left panel in **Fig. 5a**). $Mask_{Neuropil}$ is calculated through a simple thresholding procedure on $avgImg_{Norm}$ using $thr_{Neuropil}$. $Mask_{Soma}$ is calculated through an extended-maxima transform on $avgImg_{Norm}$, which consists of the regional maxima of the *H*-maxima transform of $avgImg_{Norm}$ (obtained by suppressing all maxima in $avgImg_{Norm}$ whose height is less than thr_{Soma}). Then, both $Mask_{Soma}$ and $Mask_{Neuropil}$ are imposed as regional minima to the image complement of $avgImg_{Norm}$. Finally, a watershed transformation is performed to obtain the ROI perimeters. The imposition of regional minima in $Mask_{Neuropil}$ prevents the watershed algorithm from finding “catchment basins” (i.e., ROIs) in unwanted regions (i.e., the neuropil). This allows the implementation of the watershed transform in imaged regions where labeled cells are spatially distributed in a sparse or scattered manner. Finally, the GUI can be used to manually curate ROIs, as previously explained.

Calculation of relative fluorescence variation

The data sanity test discards ROIs whose fluorescence signal is too low and/or presents artifactual fluorescence traces. For the detection of these artifacts, the module first calculates for each ROI a smooth estimate of its slowly varying fluorescence baseline (F_{smooth}). The latter is calculated using a running-window average of the 8th percentile of the ROI’s fluorescence[1], with a time window 40 times larger than the decay time constant of the calcium reporter (τ). This procedure results in a F_{smooth} that robustly tracks the ROI’s basal fluorescence level, without being affected by the fast activation transients. Thus, if an ROI is associated with a F_{smooth} that shows sudden variations exceeding a user-selected threshold, the ROI is discarded (in practice, this eliminates ROIs of unhealthy neurons or healthy neurons that move in or out of focus during the imaging session). As previously explained, the user can then choose to use F_{smooth} as an estimate of *F0* in the calculation of the $\Delta F/F0$.

The module for detection of assemblies

Besides *PCA-promax*, this module allows to cluster data using *k-means* and *hierarchical clustering* algorithms.

Briefly, *k-means* partitions data in a fixed number of clusters *k*, defined by the user. It involves randomly selecting *k* initial centroids and assigning each point (e.g., each neuron) to their closest centroids, thus forming *k* preliminary clusters. The centroids are then updated according to the points in the clusters, and this process continues until the points stop changing their clusters (i.e., convergence of the centroids). Typically, the clusters defined by *k-means* are highly independent and uncorrelated (i.e., they present low inter-cluster correlations).

On the other hand, (agglomerative) *hierarchical clustering* groups data by creating a cluster tree or dendrogram. The tree represents a multilevel hierarchy, where clusters at one level are joined as clusters at the next level. First, the similarity between every pair of variables (e.g., neuronal fluorescence traces) is calculated. Then, these similarities are used to determine the proximity of variables to each other. Variables are successively paired into binary clusters, and newly formed clusters are grouped into larger clusters in a bottom-up manner, until a hierarchical tree is formed. Finally, clustering is performed by determining where to cut the hierarchical tree, and assigning all the objects below each cut to a single cluster.

For both *k-means* and *hierarchical clustering,* the user can choose to cluster either the original z-scored dataset or the dimensionality-reduced dataset obtained through PCA. In contrast to *PCA-promax*, for both *k-means* and *hierarchical clustering*, a number of important parameters have to be set. The user must choose between using a euclidean or a pair-wise correlation metric to calculate distances between variables. For *hierarchical clustering* only, the user must choose if the distance between two clusters is defined by the shortest or longest distance between two points in each cluster (*single* and *complete* linkage clustering, respectively). Being classic methods, the particularities of clustering with these metrics and linkages has been extensively reviewed[2,3]. Finally, to obtain the final clustering for both methods, the user has to set the total number of clusters to look for in the data (*k* for *k-means*, and the smallest height at which a horizontal tree cut leaves *k* clusters for *hierarchical clustering*).

The assemblies module also calculates the time series of the assemblies' significant activations. For this, it uses a matching index, *MI*[4–6]. The *MI* is defined as

$$MI_{ij}=2\frac{|Pat_i \cap Pat_j|}{|Pat_i|+|Pat_j|} \qquad (2)$$

where Pat_i is the binary activity pattern of imaging frame *i,* and Pat_j is the binary target pattern of assembly *j* (i.e., binary *N* x 1 vectors representing the complete population of *N* ROIs, with ones indicating active ROIs and zeros indicating those inactive). Norms are equal to the number of ones in each vector. The *MI* quantifies the proportion of ROI activations that are common to both patterns with respect to the total number of activations present in both patterns. It is valued between 0 (no overlap in activations) and 1 (perfect overlap in activations). To estimate the significance of the assemblies' *MI*s over the course of the experiment, the

algorithm uses the hypergeometric distribution. Under the null hypothesis of independent ROI activations, this is a discrete distribution that describes the probability of having k "hits" with n target ROI activations in a population of N ROIs, showing K activations at a given moment. Therefore, it allows estimation of the probability of observing a given activation match by chance, with ROIs independently activated. In step 47 the user can select the threshold p-value to consider an assembly activation significant.

REFERENCES

1. Dombeck DA, Khabbaz AN, Collman F, Adelman TL, Tank DW. Imaging large-scale neural activity with cellular resolution in awake, mobile mice. Neuron. 2007;56: 43–57. doi:10.1016/j.neuron.2007.08.003

2. Izenman AJ. Modern Multivariate Statistical Techniques: Regression, Classification, and Manifold Learning. Springer Texts in Statistics. 2008. doi:10.1007/978-0-387-78189-1

3. Hastie, Trevor, Tibshirani, Robert, Friedman J. The Elements of Statistical Learning The Elements of Statistical Learning Data Mining, Inference, and Prediction, Second Edition. Springer series in statistics. 2009. doi:10.1007/978-0-387-84858-7

4. Romano SA, Pietri T, Pérez-Schuster V, Jouary A, Haudrechy M, Sumbre G. Spontaneous neuronal network dynamics reveal Circuit's Functional Adaptations for Behavior. Neuron. 2015;85: 1070–1085. doi:10.1016/j.neuron.2015.01.027

5. Hilgetag C, Kötter R, Stephan K, Sporns O. Computational methods for the analysis of brain connectivity. In: Ascoli G, editor. Computational Neuroanatomy–Principles and Methods. Totowa: Humana Press; 2002. pp. 295–335.

6. Sporns O, Honey C, Kötter R. Identification and classification of hubs in brain networks. PLoS One. 2007; doi:10.1371/Citation
