# Supplementary material for: An integrated calcium imaging processing toolbox for the analysis of neuronal population dynamics
Source: PLoS Comput Biol. 2017 Jun 7;13(6):e1005526. doi: 10.1371/journal.pcbi.1005526 (PMC5479595; doi:10.1371/journal.pcbi.1005526)
Supplement: S2 Text — (PDF) [file pcbi.1005526.s002.pdf]

COMPARISON TO OTHER METHODS

ROI segmentation

ROIs can be determined with algorithms designed to detect either morphological[1–3] or activity[4–6] features (e.g., pixels forming round fluorescent spots, or temporally correlated pixels, respectively). These approaches have different advantages and shortcomings. Clearly, the information embedded in the temporal dynamics of fluorescence can be exploited to obtain ROIs. However, activity-based methods are usually prone to target highly active neurons, whose signal-to-noise ratios (SNRs) are strongest. One popular activity-based approach uses independent component analysis (ICA) to parse the multi-dimensional signal into a combination of sparse and statistically independent signals[4]. However, this algorithm becomes impractical for large fields of view with several hundred ROIs, and assuming statistical independence (i.e., decorrelation) of signals can lead to an incorrect segmentation of single-neuron ROIs. Similarly, methods that aggregate correlated pixels[6] may fuse adjacent temporally correlated neurons (indeed, neighboring neurons in the mouse cortex tend to be more correlated than distant ones[7]). This is particularly problematic for studies of neuronal coding, and clearly limiting when investigating neuronal activity correlations (e.g., when looking for neuronal assemblies).

On the other hand, morphological-based approaches are computationally less intensive and, most importantly, unbiased with respect to the neurons' activity patterns. The caveat of this approach is that it may result in relatively imprecise ROI perimeters, and could potentially miss ROIs with low baseline fluorescence in weakly labeled imaged regions (for example, due to the spatially non-uniform staining of synthetic dye injections).

Our toolbox implements a more conservative morphological-based algorithm. To avoid segmentation problems of the imaged optical planes with uneven fluorescent labeling, the segmentation is performed on a spatially normalized image $avgImg_{Norm}$ of the acquired optical plane (see *ROI segmentation* in *The pre-processing module*). Importantly, the morphological algorithms presented here performed very well in the different preparations and the techniques studied (**Fig. 2**). Nevertheless, the GUI allows the user to skip the automatic ROI detection. In this case, the ROIs can be drawn manually, or a hexagonal grid of ROIs of a desired size can be implemented. Furthermore, the pipeline allows the import of ROIs obtained with other methods, such as the promising spatio-temporal hybrid approaches that are emerging[8], which seems to perform remarkably well in demixing fluorescent signals from potentially overlapping neuronal sources.

Inference of neuronal activations from fluorescence transients

Calcium imaging is usually performed to monitor neuronal spiking activity. However, inferring spikes from fluorescence fluctuations is a complicated computational problem, due to the complex relationship between these two variables. The reporter's fluorescence is a non-linear function of intracellular calcium, and the latter is also non-linearly related to the recent neuronal activity history. However, different fluorescence deconvolution or template matching methods have been proposed to infer estimates of spike trains and spike-

rate dynamics[8–11]. Despite not accounting for the non-linear spikes-fluorescence coupling, these algorithms have been reported to be fairly effective. Nevertheless, their performance critically depends on many experimental factors, requiring parameter tuning that usually involves electrophysiological recordings. Moreover, neurons show diverse relationships between the number of spikes fired and their fluorescence changes[12], therefore the parameters obtained for one set of neurons will not necessarily apply to other sets of neurons or experiments. Hence, we opted to keep signal filtering as minimal as possible, and implemented algorithms to detect significant fluorescent transients that can confidently be associated with neuronal activity. Importantly, as in deconvolution and template matching methods, our framework allows the extraction of fluorescence transients whose temporal dynamics are compatible with the calcium reporter biophysics (i.e., its decay time constant, τ). Revealing these significant transients is particularly important for detecting neuronal activations in behaving animals on a single-trial basis, where trial-averaging is precluded or sub-optimal[13], especially when working with low SNR signals.

Detection of functional assemblies

The identification of assemblies in large neuronal datasets is still an open problem in neuroscience. Template-matching studies have been successful in revealing the activation dynamics of a predefined population pattern[14]. These are supervised learning algorithms, since they work on labeled data (i.e., the given pattern of interest). However, to reveal hidden structure from unlabeled data, unsupervised learning algorithms are necessary. Between these approaches, we can highlight pioneering studies seeking to assess the presence of higher-order (i.e., higher than pair-wise) correlations in neuronal activity[15,16]. However, these approaches only work for triplets in sets of a few tens of neurons, and did not identify the neurons that participated in the correlations. More recently, statistical models have been used to study the prevalence of these high-order correlations in larger networks[17–21]. On the other hand, shuffling methods developed to infer the significance of repeated firing sequences were recently tested on electrophysiological recordings of ~100 neurons[22,23]. However, these algorithms rely on temporally precise coordination across neurons and, to our knowledge, have never been applied to imaging studies. Promisingly, others[24–26] have applied PCA to successfully isolate assemblies of correlated neurons, by clustering neurons according to their loadings in each principal component (PC). Importantly, the use of the Marčenko–Pastur distribution introduced by Peyrache and colleagues[24] (equation 1) allows the use of analytical and reliable statistics to infer the significantly correlated signal in the data, instead of the surrogate methods implemented in previous studies[15,16,22,23,27]. This is important, since there is still no clear agreement on which statistical features should be preserved in these control surrogate datasets.

Despite these important advances, because PCA represents the data in a space of orthogonal PCs, using PCs to delineate assemblies is misleading when neurons participate in different functional assemblies[28]. The *PCA-promax* algorithm thus further extends this work, relaxing the orthogonality condition and using the *promax*-rotated PCs to isolate assemblies. In this way, the algorithm is capable of detecting non-exclusive assemblies that share ROIs. Furthermore, this approach is data-driven, without the need for any parameter

fine-tuning. In contrast, two of the most popular unsupervised clustering algorithms used in the field, *k-means clustering* and *hierarchical clustering* (both also implemented in this toolbox), rely on the definition of key parameters (i.e., the total number of clusters and a distance threshold for the cluster tree, respectively). Furthermore, they bind members (e.g., neurons) to exclusive and non-overlapping clusters, which is problematic considering the distributed coding of the brain, as previously discussed. Nevertheless, both *k-means* and *hierarchical* clustering (and their variants) have previously been successfully used to detect functional neuronal clusters in imaging experiments[6,29–33]. Remarkably, as we have previously shown[34], the *PCA-promax* algorithm consistently and significantly outperforms both of these methods, as quantified by two standard cluster quality indexes: 1) the *Davies-Bouldin* index, which measures the ratio of the within-cluster scatter and the between-cluster separation; 2) the *normalized Hubert's Γ* index, which quantifies the agreement between the observed pair-wise neuronal correlations and the ideal case, in which pairs in assemblies have identical activities and pairs from different assemblies are uncorrelated. This improvement is most probably due to the fact that while both *k-means* and *hierarchical clustering* group variables according only to pair-wise distance measures (e.g., pair-wise correlation coefficients, euclidean distances, etc), PCA is designed to reveal the global underlying correlational structure of the dataset through an eigenvector-based multivariate analysis. Furthermore, this improvement could also be related to the previously mentioned fact that *k-means* and *hierarchical clustering* seek to detect independent or hierarchically organized clusters, respectively (see *The module for detection of assemblies*). While the former clustering feature is clearly not in agreement with the distributed nature of brain processing, the latter hierarchical character may be consistent with the hierarchical organization across brain regions, but is probably not compatible with the functional organization at the neuronal sub-circuit level. Importantly, since the *PCA-promax* method involves a dimensionality reduction procedure, it is effective in processing large datasets, as demonstrated by the detection of biologically relevant assemblies in whole-brain zebrafish light-sheet imaging experiments (~ 40,000 ROIs, **Fig. 4**). To our knowledge, few studies[6,32,35] have attempted to perform clustering in these large-scale imaging datasets, and they have all used *k-means* clustering.

As discussed above, functional clustering of neuronal data is a complex mathematical procedure still under development, and the *PCA-promax* method for detecting assemblies has limitations. The algorithm relies on PCA, which implements linear data transformations. Thus, non-linear neuronal activity correlations (i.e., when plotting the activity of a neuron against another neuron produces a curved cloud of points) could result in spurious assemblies. Most multivariate statistical analysis methods currently applied are also linear, but those that rely on manifold learning algorithms[36,37] and network theory[27] could accommodate other non-linear measures of activity similarity. Furthermore, when using PCA, there is no guarantee of a straightforward interpretation of the PCs obtained in terms of biological or experimental variables. In principle, this could be improved with the recently proposed demixed PCA technique[38], which reduces the dimensionality of the data, taking into account task parameters (e.g., sensory or motor variables). Furthermore, increasingly popular non-negative matrix factorization techniques[39], which unlike PCA impose a positivity constraint to its components, may be more biologically interpretable in terms of positive neuronal activations. Finally, the *PCA-promax* method is particularly suited to delineate discrete clusters of

ROIs that are engaged in brief events of collective activation. Thus, for population activations that present a continuous and long-lasting evolution in time (e.g., propagating spatial waves, oscillations), this algorithm would tend to discretize these dynamic phenomena in distinct patterns. For example, a wave would be separated in a progression of distinct assemblies that represent different instances of the moving wavefront. Therefore, particular care should be taken in the interpretation of the results in these kind of scenarios. Nevertheless, this is not a specific problem of the *PCA-promax* algorithm, since time-evolving patterns are usually problematic for all clustering methods.

Comparison to other toolboxes

There are few software packages available for a comprehensive analysis of calcium imaging data, despite several recent and important developments[8,40,41]. Here, we briefly describe these available toolboxes and compare them to the one presented here.

These available toolboxes are implemented in *Matlab*[8,40] and *Python*[8,41] programs, and they are all focused on pre-processing analysis of imaging data. They include within-plane motion correction for laser scanning microscopy[41], image segmentation to obtain neuronal ROIs, extraction of raw fluorescence signals[8,40,41], and signal denoising and spike deconvolution[8,40]. In particular, the most salient feature of the Tomek *et al.* package is its accelerated cell morphology-based segmentation algorithm, which can detect ~100 ROIs in 256x256 pixel images in a few milliseconds, opening the possibility for online analysis. On the other hand, Pnevmatikakis *et al.* use a powerful spatio-temporal analysis framework based on constrained non-negative matrix factorization, capable of demixing spatially overlapping neurons, which may prove important for tissue densely packed with somata.

Like the above described packages, this toolbox also performs data pre-processing and, as shown here, can be efficiently applied to different fluorescent reporters, imaging techniques, and imaged regions (**Figs. 2** and **5**). In addition, the present toolbox also covers the correction for neuropil signal contamination, mapping of functional neuronal responses and the analysis of population activity dynamics. Thus, this is the first protocol for a toolbox that covers the complete workflow from raw data to the automatic display of results. For example, Tomek *et al.* presented a supporting module to export fluorescence data along with stimulation time tags, which the user could in turn use to additionally develop a mapping procedure of functional responses. Here, we present an interactive response analysis module capable of automatically producing publication-quality figures, rich in interpretable functional information (**Fig. 6**). Furthermore, our package performs automatic cluster analysis for the detection of neuronal assemblies, even in whole-brain data (Pnevmatikakis *et al.* only pre-processed and segmented this kind of large-scale data). Finally, it contains modules for interactive and exploratory analysis of the neuronal population activity in the context of experimental and/or behavioral events, which can be extremely useful when dealing with such complex and multivariate datasets.

With respect to its implementations, the toolbox is designed in a flexible manner that allows importing data from other sources, and using separate modules in an autonomous way, bypassing preceding modules if

desired. No previous coding experience is required for its use, since interaction with the toolbox is implemented through diverse graphical user interfaces (GUIs). On the other hand, command-based packages[41] require more coding expertise, but can be run in batch mode without user interaction to automatically process collections of data. Nevertheless, complete automatization typically comes at the expense of a higher rate of data-processing errors, compared to pipelines that incorporate manual curation.

REFERENCES

1. Peron SP, Freeman J, Iyer V, Guo C, Svoboda K. A Cellular Resolution Map of Barrel Cortex Activity during Tactile Behavior. Neuron. 2015;86: 783–799. doi:10.1016/j.neuron.2015.03.027
2. Chen T-W, Wardill TJ, Sun Y, Pulver SR, Renninger SL, Baohan A, et al. Ultrasensitive fluorescent proteins for imaging neuronal activity. Nature. 2013;499: 295–300. doi:10.1038/nature12354
3. Miller J-EK, Ayzenshtat I, Carrillo-Reid L, Yuste R. Visual stimuli recruit intrinsically generated cortical ensembles. Proc Natl Acad Sci. 2014;111: E4053-61. doi:10.1073/pnas.1406077111
4. Mukamel EA, Nimmerjahn A, Schnitzer MJ. Automated analysis of cellular signals from large-scale calcium imaging data. Neuron. 2009;63: 747–60. doi:10.1016/j.neuron.2009.08.009
5. Smith SL, Häusser M. Parallel processing of visual space by neighboring neurons in mouse visual cortex. Nat Neurosci. 2010;13: 1144–9. doi:10.1038/nn.2620
6. Portugues R, Feierstein CE, Engert F, Orger MB. Whole-Brain Activity Maps Reveal Stereotyped , Distributed Networks for Visuomotor Behavior. Neuron. 2014;81: 1328–1343. doi:10.1016/j.neuron.2014.01.019
7. Smith M a, Kohn A. Spatial and temporal scales of neuronal correlation in primary visual cortex. J Neurosci. 2008;28: 12591–603. doi:10.1523/JNEUROSCI.2929-08.2008
8. Pnevmatikakis EA, Soudry D, Gao Y, Machado TA, Merel J, Pfau D, et al. Simultaneous Denoising, Deconvolution, and Demixing of Calcium Imaging Data. Neuron. 2016;89: 299. doi:10.1016/j.neuron.2015.11.037
9. Yaksi E, Friedrich RW. Reconstruction of firing rate changes across neuronal populations by temporally deconvolved Ca2+ imaging. Nat Methods. 2006;3: 377–383. doi:10.1038/NMETH874
10. Vogelstein JT, Packer AM, Machado T a, Sippy T, Babadi B, Yuste R, et al. Fast nonnegative deconvolution for spike train inference from population calcium imaging. J Neurophysiol. 2010;104: 3691–3704. doi:10.1152/jn.01073.2009
11. Grewe B, Langer D, Kasper H, Kampa BM, Helmchen F. High-speed in vivo calcium imaging reveals neuronal network activity with near-millisecond precision. Nat Methods. 2010;7: 399–405. doi:10.1038/nmeth.1453
12. Lin BB-J, Chen TT-W, Schild D. Cell type-specific relationships between spiking and [Ca2+]i in neurons of the Xenopus tadpole olfactory bulb. J Physiol. 2007;582: 163–175. doi:10.1113/jphysiol.2006.125963
13. Churchland MM, Yu BM, Sahani M, Shenoy K V. Techniques for extracting single-trial activity patterns from large-scale neural recordings. Curr Opin Neurobiol. 2007;17: 609–18. doi:10.1016/j.conb.2007.11.001

14. Louie K, Wilson MA. Temporally structured replay of awake hippocampal ensemble activity during rapid eye movement sleep. Neuron. 2001;29: 145–156. doi:10.1016/S0896-6273(01)00186-6

15. Abeles M, Gerstein GL. Detecting spatiotemporal firing patterns among simultaneously recorded single neurons. J Neurophysiol. 1988;60: 909–24.

16. Abeles M, Gat I. Detecting precise firing sequences in experimental data. J Neurosci Methods. 2001;107: 141–154. doi:10.1016/S0165-0270(01)00364-8

17. Schneidman E, Berry MJ, Segev R, Bialek W. Weak pairwise correlations imply strongly correlated network states in a neural population. Nature. 2006;440: 1007–12. doi:10.1038/nature04701

18. Shlens J, Field GD, Gauthier JL, Grivich MI, Petrusca D, Sher A, et al. The Structure of Multi-Neuron Firing Patterns in Primate Retina. J Neurosci. 2006;26: 8254–8266. doi:10.1523/JNEUROSCI.1282-06.2006

19. Roudi Y, Nirenberg S, Latham PE. Pairwise maximum entropy models for studying large biological systems: When they can work and when they can't. PLoS Comput Biol. 2009;5. doi:10.1371/journal.pcbi.1000380

20. Ohiorhenuan IE, Mechler F, Purpura KP, Schmid AM, Hu Q, Victor JD. Sparse coding and high-order correlations in fine-scale cortical networks. Nature. 2010;466: 617–21. doi:10.1038/nature09178

21. Ganmor E, Segev R, Schneidman E. Sparse low-order interaction network underlies a highly correlated and learnable neural population code. Proc Natl Acad Sci U S A. 2011;108: 9679–84. doi:10.1073/pnas.1019641108

22. Gansel KS, Singer W. Detecting Multineuronal Temporal Patterns in Parallel Spike Trains. Front Neuroinform. 2012;6: 1–16. doi:10.3389/fninf.2012.00018

23. Picado-Muiño D, Borgelt C, Berger D, Gerstein G, Grün S. Finding neural assemblies with frequent item set mining. Front Neuroinform. 2013;7: 9. doi:10.3389/fninf.2013.00009

24. Peyrache A, Benchenane K, Khamassi M, Wiener SI, Battaglia FP. Principal component analysis of ensemble recordings reveals cell assemblies at high temporal resolution. J Comput Neurosci. 2009;29: 309–25. doi:10.1007/s10827-009-0154-6

25. Peyrache A, Khamassi M, Benchenane K, Wiener SI, Battaglia FP. Replay of rule-learning related neural patterns in the prefrontal cortex during sleep. Nat Neurosci. 2009;12: 919–926. doi:10.1038/nn.2337

26. Benchenane K, Peyrache A, Khamassi M, Tierney PL, Gioanni Y, Battaglia FP, et al. Coherent Theta Oscillations and Reorganization of Spike Timing in the Hippocampal- Prefrontal Network upon Learning. Neuron. 2010;66: 921–936. doi:10.1016/j.neuron.2010.05.013

27. Humphries MD. Spike-train communities: finding groups of similar spike trains. J Neurosci. 2011;31: 2321–36. doi:10.1523/JNEUROSCI.2853-10.2011

28. Lopes-dos-Santos V, Conde-Ocazionez S, Nicolelis M a L, Ribeiro ST, Tort ABL. Neuronal assembly detection and cell membership specification by principal component analysis. PLoS One. 2011;6: e20996. doi:10.1371/journal.pone.0020996

29. Ozden I, Sullivan MR, Lee HM, Wang SS-H. Reliable coding emerges from coactivation of climbing fibers in microbands of cerebellar Purkinje neurons. J Neurosci. 2009;29: 10463–73. doi:10.1523/JNEUROSCI.0967-09.2009

30. Dombeck DA, Graziano MS, Tank DW. Functional clustering of neurons in motor cortex determined by cellular resolution imaging in awake behaving mice. J Neurosci. 2009;29: 13751–60. doi:10.1523/JNEUROSCI.2985-09.2009

31. Bonifazi P, Goldin M, Picardo MA, Jorquera I, Cattani A, Bianconi G, et al. GABAergic hub neurons orchestrate synchrony in developing hippocampal networks. Science. 2009;326: 1419–24. doi:10.1126/science.1175509

32. Panier T, Romano SA, Olive R, Pietri T, Sumbre G, Candelier R, et al. Fast functional imaging of multiple brain regions in intact zebrafish larvae using Selective Plane Illumination Microscopy. Front Neural Circuits. 2013;7: 65. doi:10.3389/fncir.2013.00065

33. Bathellier B, Ushakova L, Rumpel S. Discrete neocortical dynamics predict behavioral categorization of sounds. Neuron. 2012;76: 435–49. doi:10.1016/j.neuron.2012.07.008

34. Romano SA, Pietri T, Pérez-Schuster V, Jouary A, Haudrechy M, Sumbre G. Spontaneous neuronal network dynamics reveal Circuit's Functional Adaptations for Behavior. Neuron. 2015;85: 1070–1085. doi:10.1016/j.neuron.2015.01.027

35. Freeman J, Vladimirov N, Kawashima T, Mu Y, Sofroniew NJ, Bennett D V., et al. Mapping brain activity at scale with cluster computing. Nat Methods. 2014;11: 941–950. doi:10.1038/nmeth.3041

36. Roweis ST, Saul LK. Nonlinear dimensionality reduction by locally linear embedding. Science. 2000;290: 2323–2326. doi:10.1126/science.290.5500.2323

37. Stopfer M, Jayaraman V, Laurent G. Intensity versus identity coding in an olfactory system. Neuron. 2003;39: 991–1004. doi:10.1016/j.neuron.2003.08.011

38. Kobak D, Brendel W, Constantinidis C, Feierstein CE, Kepecs A, Mainen ZF, et al. Demixed principal component analysis of neural population data. Elife. 2016;5: 1–37. doi:10.7554/eLife.10989

39. Lee DD, Seung HS. Learning the parts of objects by non-negative matrix factorization. Nature. 1999;401: 788–91. doi:10.1038/44565

40. Tomek J, Novak O, Syka J. Two-Photon Processor and SeNeCA: a freely available software package to process data from two-photon calcium imaging at speeds down to several milliseconds per frame. J Neurophysiol. 2013;110: 243–56. doi:10.1152/jn.00087.2013

41. Kaifosh P, Zaremba JD, Danielson NB, Losonczy A. SIMA: Python software for analysis of dynamic fluorescence imaging data. Front Neuroinform. 2014;8: 80. doi:10.3389/fninf.2014.00080
