# Supplementary material for: An integrated calcium imaging processing toolbox for the analysis of neuronal population dynamics
Source: PLoS Comput Biol. 2017 Jun 7;13(6):e1005526. doi: 10.1371/journal.pcbi.1005526 (PMC5479595; doi:10.1371/journal.pcbi.1005526)
Supplement: S3 Text — (PDF) [file pcbi.1005526.s003.pdf]

INSTALLATION INSTRUCTIONS

Equipment

The pipeline can be run on any desktop computer, but we recommend multicore computers for shorter computing times, as several algorithms allow for parallelization (especially for the pre-processing module). For analysis of light-sheet imaging data, we used a computer cluster of 328 CPUs based on a HTCondor parallelization system. Imaging videos of long experiments can result in file sizes of several gigabytes (GB), and therefore we recommend 64-bit architectures with at least 12 GB of RAM. We also recommend screen resolutions of at least 1024 x 768 pixels for a better experience when visualizing data and using user interfaces.

A *Matlab* installation (MathWorks) is required, including the following toolboxes: Curve Fitting, Image Processing, Statistics and Machine Learning, and Parallel Computing. This list of toolbox requirements refers to the full processing pipeline; specific modules need different subsets of these toolboxes. We verified compatibility specifically for *Matlab* versions R2010a, R2011b, R2014a, and 2015b, but our code should, in principle, be compatible with any later version.

Computer operating systems: any version of Windows, Linux or Mac OS X compatible with the *Matlab* installation.

Software setup

Installation of the toolbox. Download the analysis toolbox from www.zebrain.biologie.ens.fr/codes. It consists of a *zip* file that contains all the *Matlab* source code and a *Readme.pdf* file that explains all the relevant variables used during the pipeline (this file is vital if the user wishes to adapt or further develop the toolbox). It also contains test data for a study case. Double-click on the downloaded toolbox *zip* file to extract its contents. Add the toolbox to *Matlab* path. For this, open *Matlab* and type the command:

addpath(genpath('Extracted_Folder'))

where Extracted_Folder is the name of the toolbox unzipped folder.

Install *ImageJ* and its *Template Matching and Slice Alignment* plugin. Download *ImageJ* from http://imagej.nih.gov/ij/ and the *Template Matching and Slice Alignment* from sites.google.com/site/qingzongtseng/template-matching-ij-plugin and install them following the installation instructions of the corresponding websites.
