# Supplementary figures and images for: An integrated calcium imaging processing toolbox for the analysis of neuronal population dynamics
Source: PLoS Comput Biol. 2017 Jun 7;13(6):e1005526. doi: 10.1371/journal.pcbi.1005526 (PMC5479595; doi:10.1371/journal.pcbi.1005526)

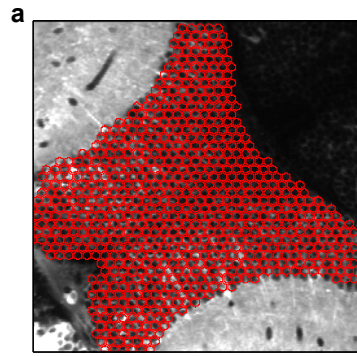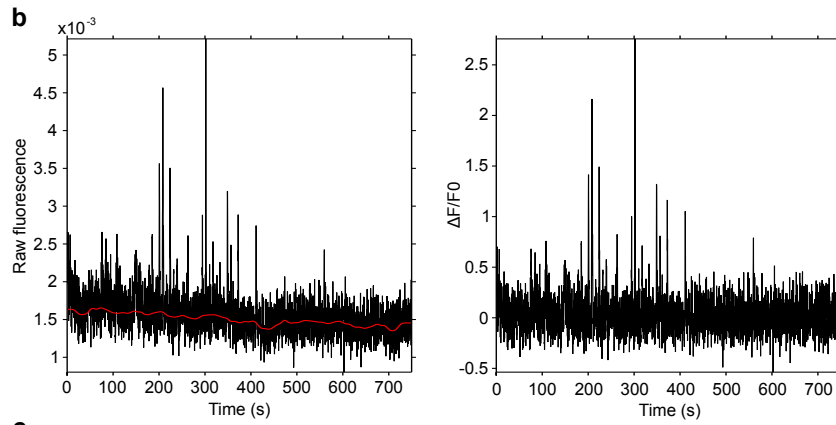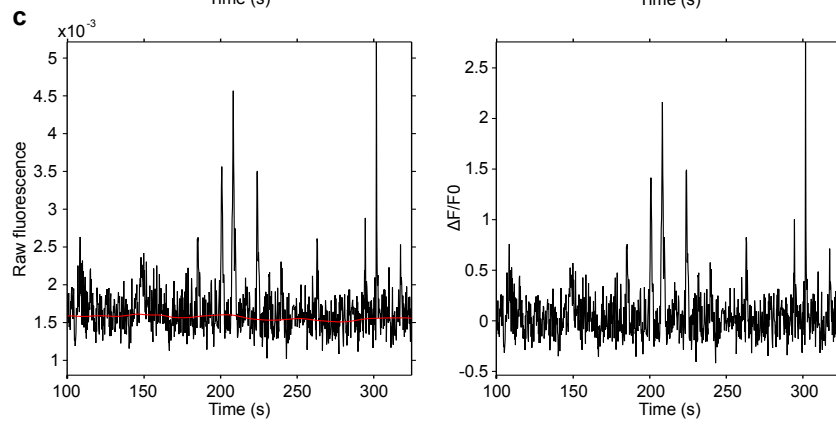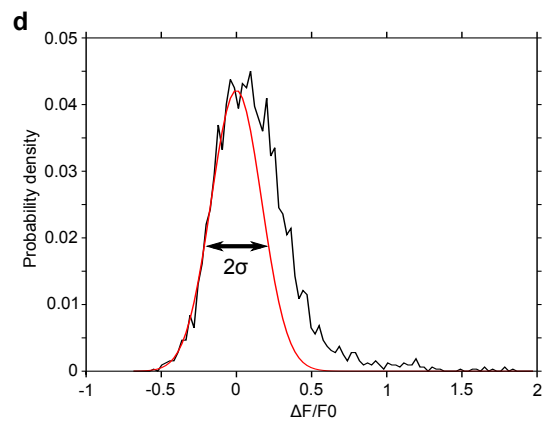

Supplement: S1 Fig — (A) Red, hexagonal grid of 6 mm-diameter, over an imaged optical section of optic tectum of a zebrafish larva pan-neuronally expressing GCaMP3. The region covered by the grid is defined with a user-drawn mask. (B) Left, raw fluorescence of a ROI (black) and the estimated Fsmooth. Right, ΔF/F0 obtained using Fsmooth as F0. Note how slow fluctuations are removed, producing a stable ΔF/F0. (C) Zoom of A. Note how follows slow fluorescence variations, ignoring the fast, neuronal activity related fluorescence transients. (D) Estimation of the baseline fluorescence noise (σ) of a ROI. Black, normalized histogram of the ROI's ΔF/F0; red, Gaussian fit to the negative fluorescence ΔF/F0. (PDF) [file pcbi.1005526.s004.pdf]

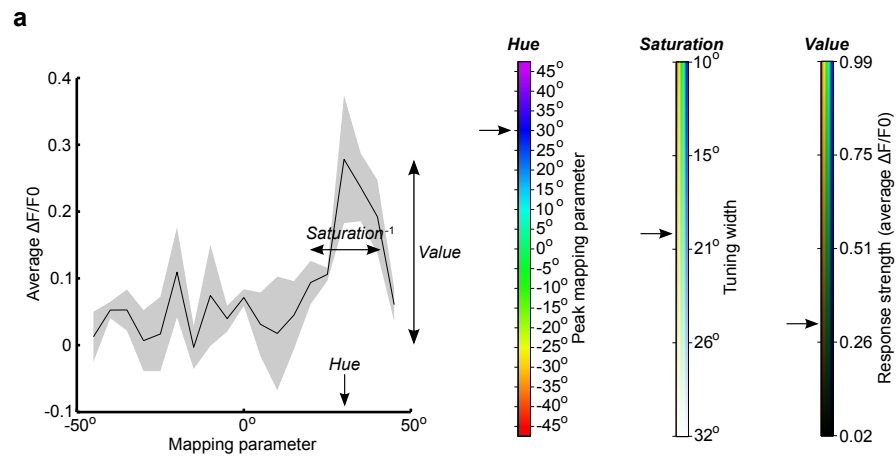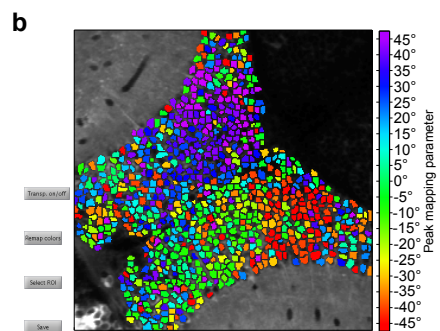

Supplement: S2 Fig — (A) Schema illustrating the definition of the hue, saturation and value to visually represent ROI responses in a color-code. Left, ROI tuning curve. Arrows indicate the particular hue, saturation and value of this ROI. Right, colorbars representing the range of hue, saturation and value for all the imaged ROIs. Arrows indicate the color-code parameters for the ROI tuning curve shown in the left. (B) Display of neuronal responses only representing preferred stimulus (i.e., the peak mapping parameter). Same as Fig 5, but disabling saturation and value channels, thus only representing preferred stimulus on the hue channel. The noisier image obtained underscores the utility of additionally representing the neuronal selectivity and response strength. (PDF) [file pcbi.1005526.s005.pdf]
